# Redesign of the *Chlamydomonas reinhardtii* Q_B_ binding niche reveals photosynthesis works in the absence of a driving force for Q_A_ ‐Q_B_ electron transfer

**DOI:** 10.1111/ppl.70008

**Published:** 2024-12-14

**Authors:** Maya D. Lambreva, Veranika Zobnina, Taras K. Antal, Violeta N. Peeva, Maria Teresa Giardi, Ivo Bertalan, Udo Johanningmeier, Olli Virtanen, Mithila Ray, Paula Mulo, Fabio Polticelli, Esa Tyystjärvi, Giuseppina Rea

**Affiliations:** ^1^ Institute for Biological Systems, National Research Council Monterotondo Stazione (RM) Italy; ^2^ Department of Sciences University Roma Tre Rome Italy; ^3^ Laboratory of integrated ecological research Pskov State University Pskov Russia; ^4^ Bulgarian Academy of Sciences Institute of Plant Physiology and Genetics Sofia Bulgaria; ^5^ Biosensor Srl Formello Rome Italy; ^6^ Institute of Crystallography, National Research Council Monterotondo Stazione (RM) Italy; ^7^ Institut für Pflanzenphysiologie, Martin‐Luther‐Universität Halle‐Wittenberg Halle (Saale) Germany; ^8^ Department of Life Technologies/Molecular Plant Biology University of Turku Turku Finland; ^9^ Department of Physics and Astronomy Vrije Universiteit Amsterdam Amsterdam The Netherlands; ^10^ National Institute of Nuclear Physics, Roma Tre Section Rome Italy

## Abstract

An in silico redesign of the secondary quinone electron acceptor (Q_B_) binding pocket of the D1 protein of Photosystem II (PSII) suggested that mutations of the F265 residue would affect atrazine binding. *Chlamydomonas reinhardtii* mutants F265T and F265S were produced to obtain atrazine‐hypersensitive strains for biosensor applications, and the mutants were indeed found to be more atrazine‐sensitive than the reference strain IL. Fluorescence and thermoluminescence data agree with a weak driving force and confirm slow electron transfer but cannot exclude an additional effect on protonation of the secondary quinone. Both mutants grow autotrophically, indicating that PSII requires strong light for optimal function, as was the case in the ancestral homodimeric reaction center.

## 1 INTRODUCTION

Photosynthetic reaction centres (RCs) come in two basic varieties (Allen & Williams, 1998, 2011; Blankenship, 2021; Gorka et al., 2021). Type I RCs include the heterodimeric Photosystem I (PSI) of oxygenic photosynthesis (Caspy & Nelson, 2018) and the homodimeric RCs of anoxygenic photosynthesis (Gisriel et al., 2017; Orf et al., 2018). All Type II RCs are heterodimeric, with homologous D1 and D2 proteins in the oxygenic PSII (Umena et al., 2011), and L and M subunits in the anoxygenic RCIIs (Deisenhofer et al. 1985). Type II RCs feature a pair of chlorophyll (Chl) or bacteriochlorophyll (BChl) molecules positioned with the chlorin ring planes roughly on top of each other, two other BChl *a* or Chl *a* molecules, two (bacterio)pheophytins and quinone acceptors Q_A_ and Q_B_ that mediate electrons to a mobile quinone pool (Allen & Williams, 1998; Sugo et al., 2022). Q_B_ is a two‐electron carrier and after full reduction and protonation to Q_B_H_2_, it is displaced by another quinone from the pool.

All monomers of the photosystems bear a weak amino acid sequence similarity, but the organization of the helices and the locations of the key cofactors are so similar in all photosystems that there is little doubt about a common origin (Grotjohann et al., 2004; Orf et al., 2018). Evolution of the photosynthetic RCs, including the timing and first host organism of oxygen evolution is a topic of debate (for review, see Hohmann‐Marriott & Blankenship, 2011; Fischer et al., 2016; Sánchez‐Baracaldo et al., 2022). Geological data have been interpreted to indicate a shift from anoxic (< 10^−5^ × present atmospheric level, PAL) to oxic atmospheric conditions (> 10^−4^ × PAL) at 3 billions of years ago (Ga; Ohmoto et al., 2006; Crowe et al., 2013), 2.45 Ga (Farquhar et al., 2000) or up to 2.2 Ga ago (Kirschvink & Kopp, 2008). The oxygen content of the atmosphere may also have oscillated in the Archaean time (Ohmoto et al., 2006; for review of the geological data, see Lyons et al., 2014). Biological evidence about the development of oxygenic photosynthesis, in turn, can be interpreted to support a model in which oxygenic evolution arose via horizontal transfer of the genes encoding one type of photosystem to an organism that already had the other type, or via horizontal transfer of genes for both photosystems to a non‐photosynthetic organism (Fischer et al., 2016). The horizontal gene transfer model has been challenged mainly on the basis of the finding that D1 and D2 are closer relatives to each other than D1 to L or D2 to M (Cardona, 2015). Thus, oxygenic and anoxygenic photosynthesis may have evolved side by side rather than sequentially (Cardona et al., 2012, 2019; Cardona, 2019). The phylogenetic history of cyanobacteria adds a further layer of complexity (Fischer et al., 2016; Sánchez‐Baracaldo et al., 2022), as molecular clock data suggest that cyanobacteria evolved 3 Ga ago (Fournier et al., 2021), whereas analysis of respiratory and photosynthetic enzymes in Cyanobacteria, including *Vampirovibronia* and *Sericytochromatia*, the non‐photosynthetic predecessors of *Oxyphotobacteria*, suggest that oxygen evolution was brought horizontally to cyanobacteria only very near to the Great Oxygenation Event at 2.45 Ga (Soo et al., 2017). Evolution of the reaction centre proteins CP43 and CP47, in turn, suggests that oxygen evolution evolved very near to the origin of life (Oliver et al., 2021), a conclusion supported by the finding that the reaction centre genes of PSII are under strong purifying selection (Ślesak et al., 2022).

Irrespective of the unknowns of the evolution of photosynthesis, the photosystems are universally dimeric, implying that they evolved from homodimeric ancestors that already performed light‐powered electron transfer (ET; Cardona et al., 2019). However, opinions differ on whether the evolution passed through a reaction centre with intermediate RCI/RCII characteristics but with two quinones (Orf et al., 2018) or whether both Type I and Type II had a homodimeric ancestor of their own (Cardona et al., 2019).

In a homodimeric Type II RC or an intermediate Type I/II quinone‐binding RC, either quinone can assume the role of Q_A_ on random basis, and a mechanism that prevents the formation of a Q_A_ˉQ_B_ˉ state via ET from the primary donor to the second quinone must be operating (Cardona et al., 2012). The quantum efficiency of a homodimeric Type II RC has been suggested to be low because there would be no driving force for ET from Q_A_ to Q_B_ (Cardona et al., 2012; Orf et al., 2018). Efficient use of light was probably not mandatory for organisms hosting a homodimeric Type II RC since most probably, as suggested by (Cardona et al., 2012), the competition for light was not a major evolutionary pressure. However, there is no experimental evidence about ET in homodimeric Type II RC.

In the present study, we modelled ET in a homodimeric reaction centre by using a genetically modified strain of the green alga *Chlamydomonas reinhardtii*. The original aim was to design PSII mutants with improved affinity for atrazine (ATZ), a herbicide binding to the Q_B_ pocket, to be used as biosensors (Erickson et al., 1989; Giardi & Pace, 2005; Rea et al., 2009). The PSIIs of the resulting D1 protein mutant strains F265S and F265T are not full models of a homodimeric reaction centre, as they are heterodimeric and the two‐electron gate at the Q_B_ site is fully functional in them. However, the light response of photosynthesis in both mutants is similar as it would be in a reaction centre lacking a driving force for ET from Q_A_ to Q_B_, as photosynthesis is very slow in low light but approaches the control rate in strong light. Biophysical characterization of the mutants agrees with the lack of a driving force but cannot exclude an additional effect on the protonation of Q_B_^−^ or Q_B_^2−^.

## 2 MATERIALS AND METHODS

### 2.1 Molecular dynamics (MD) simulations

The ATZ molecule was positioned inside the wild type (WT) Q_B_ binding pocket according to a previous docking study carried out in our lab (Rea et al., 2009). The complex was refined through energy minimization and restrained MD simulation (10 ns simulation with the position restraints applied to non‐hydrogen atoms of protein subunits). The position of ATZ within the binding pocket changed and the final structure of the complex, including hydrogen bonds, is shown in Figure S1.

The possibility to redesign the Q_B_ binding pocket in order to obtain PSII variants able to more efficiently recognize herbicide molecules was investigated through MD simulations of WT and mutant PSII in complex with the natural ligand Q_B_ and with the herbicide ATZ. In detail, 10 ns MD simulations of the wild type and two mutated variants of the PSII complex with the Q_B_ molecule or ATZ within the Q_B_ binding site were performed. Phe265 of the protein D1 was mutated to Thr or Ser, as indicated. Full methodological details of the MD simulations are reported in (Zobnina et al., 2017).

The electrostatic component of the binding energy of the ligands to PSII was calculated as the total electrostatic energy of the complex minus the sum of the electrostatic energy of the isolated components: *ΔG*_*el*_ = *G*_*el*_ *complex−*(*G*_*el*_ *PSII* + *G*_*el*_ *ligand*), where *G*_*el*_ is calculated as the sum of the solvation and coulombic contributions to electrostatic energies, calculated using the program APBS (Jurrus et al., 2018).

### 2.2 Production and growth of *C. reinhardtii* strains

The *C. reinhardtii* strain IntronLess (IL; Johanningmeier & Heiss, 1993), was used as a reference strain in this study. Its deletion mutant Del1 (Preiss et al., 2001) was biolistically transformed to harbour the substitution of phenylalanine (Phe, F) at position 265 of the D1 protein by threonine (T) or serine (S) in the F265T and F265S mutants, respectively, as previously described (Rea et al., 2011a). A schematic representation of the site‐directed mutagenesis and the sequences of the specific primers are reported in Figure S2 and Table S1, respectively. The photoautotrophic growth of the IL strain and the two D1 mutants was evaluated in High Salt (HS) medium bubbled with 2.5% CO_2_ at 23°C and the photosynthetic photon flux density (PPFD) of 80 μmol m^−2^ s^−1^, during 400 h by measuring culture optical density at 750 nm (OD_750_).

*Chlamydomonas* strains were maintained on TAP (Harris, 1989) agar plates at 24 ± 1°C and 50 μmol photons m^−2^ s^−1^ continuous illumination or grown in TAP medium under 150 rpm agitation. Mixotrophic culture growth was registered during 88 h by measuring cell number per ml (Automated Cell Counter, TC10, Bio‐Rad Laboratories, Inc), the OD_750_ and Chl content as previously described (Lambreva et al., 2013). All experiments were performed using cultures in mid‐exponential growth phase (OD_750_ = 0.6 ± 0.05).

### 2.3 Growth of *Synechocystis sp.* PCC 6803

The construction of the PD strain (hosting deletion, Δ(225–239), of the D‐E loop of the D1 protein coded by the *PSBA2* gene) and its basic properties in comparison to the control strain (Antibiotic Resistance, AR; a strain from which the *PSBA1* and *PSBA3* genes have been inactivated with antibiotic resistance cassettes) have been earlier described (Mulo et al., 1997). PD and AR strains were grown on Agar plates at PPFD 30 μmol m^−2^ s^−1^ in BG‐11 growth medium supplemented with antibiotics and 3‐(3,4‐dichlorophenyl)‐1,1‐dimethylurea (DCMU) as described earlier (Mulo et al., 1997). For growth experiments, cells were first washed twice with BG‐11 growth medium to remove the antibiotics and DCMU, and then suspended in BG‐11 at the OD_750_ of 0.1. Growth experiments were done in Erlenmeyer bottles in 30 mL volume at 32°C, with constant stirring and with 1% CO_2_ in the airspace of the Algaetron (PS Instruments) growth chamber. OD_750_ was used to assay growth.

### 2.4 Chl *a* fluorescence measurements

All Chl fluorescence measurements were performed at room temperature on samples containing 10 ± 0.8 μg ml^−1^ of Chl, unless otherwise specified. Chl *a* fluorescence induction curves (*OJIP* transient) were measured from 10 min dark acclimated samples by Plant Efficiency Analyser fluorimeter (Hansatech Instruments, King's Lynn) as previously described (Lambreva et al., 2013). The *F*_*v*_ */F*_*m*_ fluorescence parameter, earlier associated with the maximum quantum yield of PSII photochemistry but recently shown to mostly reflect conformational changes in closed PSII centres upon illumination (Sipka et al. 2021), was calculated as *F*_*v*_ */F*_*m*_ = (*F*_*m*_−*F*_*0*_)/F_*m*_; the efficiency of the ET between PSII Q_A_ and Q_B_ electron acceptors was evaluated by the parameter *1−V*_*J*_, where *V*_*J*_ = (*F*_*2ms*_−*F*_*0*_)/(*F*_*m*_−*F*_*0*_) (Strasser et al., 2000). *F*_*0*_, *F*_*m*_ and *F*_*2ms*_ are the fluorescence level at 50 μs, the maximum fluorescence and the fluorescence level at 2 ms after the onset of the illumination, respectively.

Rapid light curves of estimated relative Electron Transfer Rate (*rETR*) were registered on 5 min dark acclimated samples using MULTI‐COLOR‐PAM (Heinz Walz GmbH) as previously described (Havurinne et al., 2019). The intensity settings of the measuring light (625 nm) and the 3 ms long saturating pulses were 1 and 20, respectively, and the duration of each light step of the rapid light curves was 30 s. Details on parameter calculation are available in Supplementary Information.

The decrease in Chl *a* fluorescence yield after a single turnover flash, reflecting the kinetics of Q_A_^−^ reoxidation, was measured with a double modulation fluorometer (FL2000, Photon Systems Instruments) in the 150 μs to 100 s time range. A Peltier thermocouple was used for temperature control. The samples were dark acclimated for 3 min and Q_A_^−^ re‐oxidation was measured at room temperature in presence of different concentrations (from 1 × 10^−9^ to 4 × 10^−7^ M) of ATZ (Sigma‐Aldrich, Supelco grade) dissolved in DMSO (Sigma‐Aldrich). The temperature dependency of Q_A_^−^ re‐oxidation (15–35 °C) in the presence of 20 μM DCMU was also measured in order to calculate activation parameters of the S_2_Q_A_^−^ → S_1_Q_A_ recombination reactions (Rantamäki and Tyystjärvi, 2011). The fluorescence decay was normalized to the minimum and maximum values of measured fluorescence intensity using the formula: *F = (F*_*raw*_ *−F*_*0*_ *)/(F*_*150μs*_ *−F*_*0*_), where *F* and *F*_*raw*_ are the normalized and raw fluorescence intensities, respectively, *F*_*0*_ represents the value measured before the single‐turnover flash, and *F*_*150μs*_ is the fluorescence intensity measured 150 μs after the single‐turnover flash.

### 2.5 Thermoluminescence (TL) measurements

TL was measured with a home‐built device (Zeinalov & Maslenkova, 1996) using 100 μL samples containing 500 μg ml^−1^ Chl pipetted on a Whatman filter paper placed on the instrument sample holder at 20°C. The sample holder was equipped with a resistance wire (3 Ohm/10A) for the heating and a microthermoresistor for the temperature measurements. Each sample was cooled down to 1°C and kept for 1 min at this temperature before illumination with 1, 2 or 3 saturating single turnover xenon flashes (4 J, 10 μs half‐band, 1 Hz frequency). The samples were warmed up at a constant rate of 0.5 °C s^−1^ and the TL signal rising from the S_2(3)_Q_B_^−^ charge recombination was detected from 1 to 70°C by an R943‐02 photomultiplier tube (Hamamatsu Photonics). TL arising from the S_2_Q_A_^−^ charge recombination (Q‐band) was measured from cells incubated with 10 μM DCMU for 5 min in the dark at 20°C and then excited with a single saturating flash at −5°C (Ducruet, 2013).

### 2.6 Oxygen evolution measurements

Light response curves of oxygen evolution were measured with a Clark‐type oxygen electrode (Hansatech Instruments) at 24°C and continuous stirring on samples containing 10 μg ml^−1^ Chl and 10 mM NaHCO_3_. Oxygen evolution was recorded for 30 s after 30 s exposure to different PPFDs in the range of 100–2000 μmol m^−2^ s^−1^. For the cyanobacterial strains, oxygen evolution was measured in 12–2000 μmol m^−2^ s^−1^ PPFD range in the presence of 0.4 μM 2,6‐dichlorobenzoquinone as an electron acceptor and with 0.4 μM ferricyanide to maintain the electron acceptor oxidized. The cells were suspended at OD_750_ of 0.8, Chl *a* concentration was measured afterwards with methanol extraction and the results were calculated per mg of Chl.

The kinetics of oxygen evolution induced by sequences of single turnover flashes was measured with a bare platinum oxygen electrode (Zeinalov, 2002). 100 μL cell suspension, containing 300 μg ml^−1^ Chl and incubated with 0.6 mM phenyl‐*p*‐benzoquinone, was placed on the platinum cathode (50.27 mm^2^) and covered with a cellophane membrane. The top compartment of the electrode that forms a salt bridge through the cellophane membrane and by contact to the anode, was filled with TAP medium supplemented with 100 mM KCl. Short saturating flash sequences from a xenon photoflash (t_1/2_ = 10 μs, 4 J) with 300 ms interval between flashes were used. Estimation of initial states of the Oxygen Evolution Complex (OEC) and the miss and double hit parameters was done by minimizing the mean quadratic deviation between the yields experimentally obtained and those predicted by Kok's model (Kok et al., 1970). The miss and double hit parameters and the ratio of initial states S_0_ and S_1_ were free running; initial concentrations of other S‐states were assumed to be zero. Thus, the distribution of the initial states of OEC in the dark (S_0_ and S_1_), and misses (α) and double hits (β) factors were calculated.

The sensitivity of the wild‐type and D1 mutants of *Chlamydomonas* to ATZ was evaluated by recording the oxygen evolution rate at 800 μmol m^−2^ s^−1^ in the presence of different concentrations of ATZ (from 5 × 10^−10^ to 1 × 10^−6^ M) as previously described (Husu et al., 2013). The registration started immediately after the addition of the herbicide and continued for 3 min. The inhibition of oxygen evolution induced by ATZ was calculated as:

(Eq 1) $$\mathrm{Inh},\%=\left(1-R_{\mathrm{ATR}}/R_{\text{CTRL}}\right)\times 100,$$

where *R*_*CTRL*_ and *R*_*ATR*_ are the light saturated oxygen evolution rates in absence and presence of ATZ, respectively. The inhibition constant *I*_*50*_ (ATZ concentration required for 50% inhibition of *R*) was determined by fitting the experimental dose–response curves to a typical isothermal binding equation:

(Eq 2) $$y=\left(I_{\mathrm{MAX}}\times x\right)/\left(I_{50}+x\right),$$

where *x* and *y* are the molar herbicide concentration and the percentage of inhibition of *R*, respectively, and *I*_*MAX*_ is the maximum inhibition of *R*. *I*_*50*_ and *I*_*MAX*_ are free parameters obtained by the fitting of the experimental data. Because oxygen consumption by respiration continues even if oxygen evolution ceases, the reduction of *R* exceeded 100%, which would have led to an overestimation of *I*_*50*_ achieved from a fit of the dose–response curve. Therefore, the corrected *I*_*50*_ was calculated by solving Equation 2 for y = 50% using *I*_*50*_ and *I*_*MAX*_ obtained from the fit. This procedure provided values of *I*_*50*_ comparable with our previous studies (Husu et al., 2013; Lambreva et al., 2013). The limit of detection (LOD), or lowest concentration of ATZ that may be experimentally detected with a certain confidence level, was calculated as:

(Eq 3) $$\mathrm{LOD}=\left(2.6\times \sigma \times I_{50}\right)/\left(100-2.6\times \sigma \right),$$

where σ is the mean standard error of the measurements, and *2.6 × σ* indicates the confidence interval in which there is a 99% probability of finding measurement results following the assumption of a normal distribution (Maly et al., 2005).

### 2.7 Statistical analyses and modelling

All data are means of at least three independent experiments with three technical repetitions each, unless differently specified. The differences between the reference strain and each of the D1 mutants were assessed by a non‐parametric Mann–Whitney U test for comparison of two independent samples. The statistical significance of the differences was evaluated at *p* ≤ 0.05. Models of oxygen flash sequences were compared using the Akaike Information Criterion (Akaike 1974).

The light response of PSII electron transfer was modelled with Copasi v. 4.44 (Hoops et al. 2006). The model consisted of the light‐dependent transitions Q_A_Q_B_ → Q_A_^−^Q_B_ and Q_A_Q_B_^−^ → Q_A_^−^Q_B_^−^, and reactions Q_A_^−^Q_B_ ↔ Q_A_Q_B_^−^ (forward rate constant k_AB_, backward k_BA_); Q_A_^−^Q_B_^−^ ↔ Q_A_Q_B_^2^^−^ (k_ABm_, k_BmA_); Q_A_Q_B_^2^^−^ → Q_A_^−^[Q_B‐EMPTY SITE_] (k_PROTONATION_); Q_A_^−^[Q_B‐EMPTY SITE_] → Q_A_Q_B_ (k_BINDING_); Q_A_^−^Q_B_ → Q_A_Q_B_ and Q_A_^−^Q_B_^−^ → Q_A_Q_B_^−^ (k_RECOMBINATION_). All reactions were assumed to be of first order, and the values of the rate constants of wild‐type PSII, in s^−1^, were k_AB_ = 2777, k_BA_ = 138, k_ABm_ = 300, k_BmA_ = 15, k_PROTONATION_ = 50, k_BINDING_ = 50, k_RECOMBINATION_ = 0.16. PSII with no driving force for Q_A_^−^Q_B_ electron transfer was defined as (k_BA_ = 2777, k_BmA_ = 300) and slow protonation was modelled as (k_PROTONATION_ = 6.25).

## 3 RESULTS

### 3.1 MD simulations of WT and mutant PSII with the natural ligand Q_B_ and with ATZ

**FIGURE 1:**
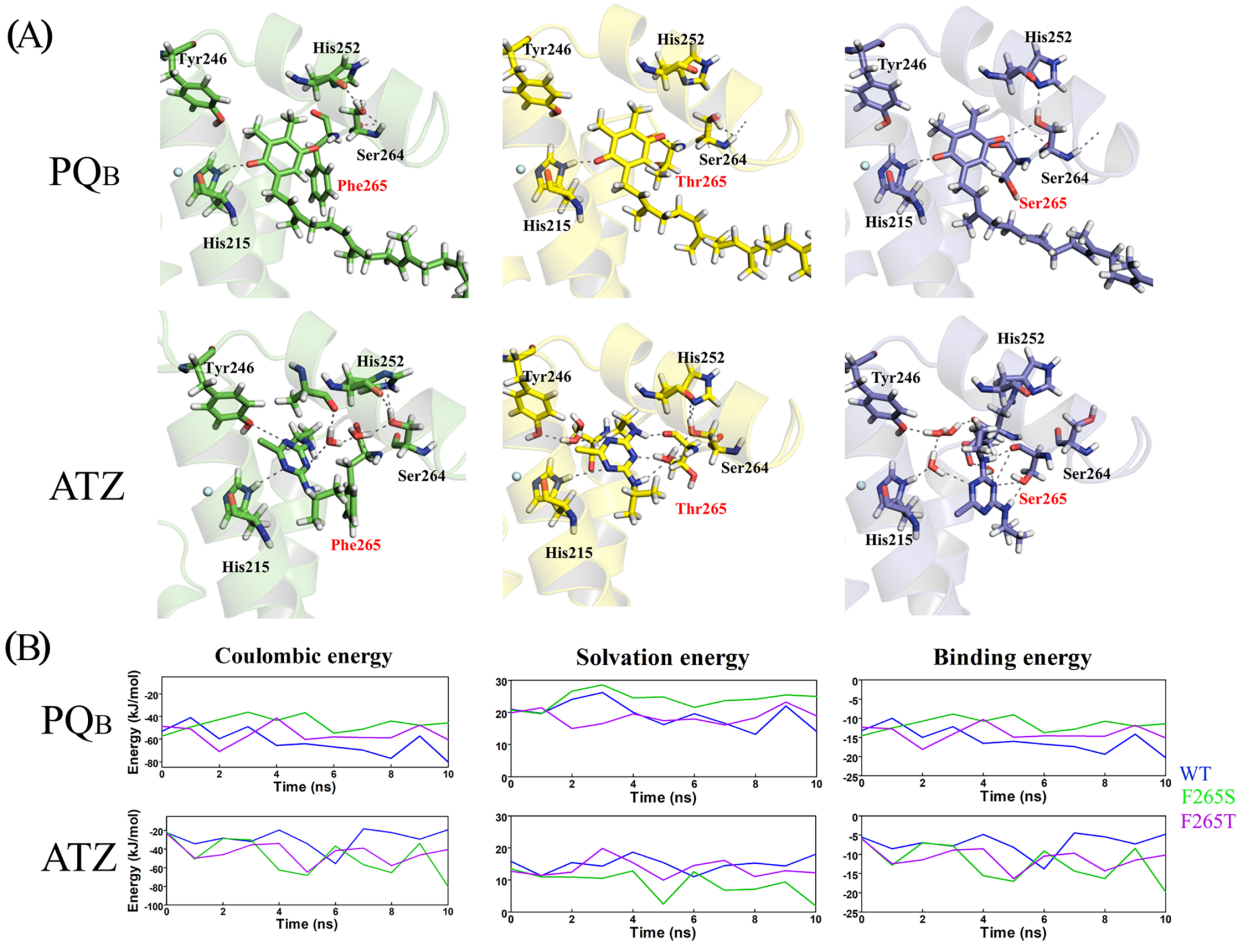
**Results of the MD simulations of WT and mutant PSII with the natural ligand Q**_B_ **and with ATZ. (**A) Schematic representation of the complexes of plastoquinone (PQ_B_) and ATZ within the Q_B_ binding pocket after 10 ns MD simulations (green – WT, purple – F265S, yellow – F265T). Hydrogen bonds are shown as dashed lines. (B) Time evolution of Coulombic energy, solvation energy and binding energy values calculated for the PSII complex with PQ_B_ and ATZ, as indicated.

The F265 residue of the D1 protein was chosen as a target of mutagenesis because docking simulations of the complex between PSII and ATZ evidenced a non‐optimal binding of the herbicide ATZ within the PSII Q_B_ binding pocket (Figure S1). MD simulations of WT and mutant PSII with the natural ligand Q_B_ and with ATZ were done to predict the properties of mutants F265T and F265S. In the simulations, the distance between the headgroup of Q_B_ and the non‐heme iron atom was 8.6–9.2 Å in the wild type, 8.6–9.8 Å in F265T and 8.8–9.6 Å in F265S (Figure S3). Additional intermolecular hydrogen bonds, also involving several water molecules, were observed in F265T and F265S, in comparison to the wild type. In particular, a hydrogen bonding network connecting the triazine ring (N3) nitrogen atom with His215, Tyr246, Ser268 and Asn266 through multiple water bridges was observed in F265S (Figure 1, panel A). ATZ remained essentially at the same distance from the non‐heme iron in WT and F265T but switched to a more distant position after 1 ns of simulation in F265S (Figure S4).

Changing F265 to T or S weakened the binding of the Q_B_ molecule (Figure 1, panel B). Mutation F265T resulted in unfavourable interactions and in F265S, both solvation and coulombic contributions to the electrostatic component of the binding energy were unfavourable. In simulations of F265S with ATZ, the contribution of the solvation energy to the binding energy was generally lower (i.e., more favourable) than in simulations with WT or F265T, while the values of the coulombic contribution were lower for both mutated complexes than for WT. As a result, the overall electrostatic component of the binding energy of ATZ was more negative (i.e., more favourable) for the mutated complexes than for WT. The electrostatic complementarity between ATZ and the surface of the Q_B_ pocket was best for F265S (Figure S5).

### 3.2 Physiological characterisation of the *Chlamydomonas* mutants

#### 3.2.1 Photoautotrophic and mixotrophic growth

F265T and F265S were produced via site‐directed mutagenesis (see Figure S2 for the details of the site‐directed mutagenesis and Table S1 for the PCR primers). The new strains grew photoautotrophically, maintaining approximately 70% (F256T) and 20% (F256S) of the growth rate of the reference strain IL (Figure S6A). Under mixotrophic conditions, the growth rates of the two mutants were approximately one half of that of IL, the difference between F256T and F256S in terms of cell proliferation rate was not pronounced (Figure S6b). Furthermore, the Chl content of the F265T and F265S strains was 48 and 42% of that of IL, respectively (Table 1, Figure S6C).

**TABLE 1:** Pigment content and PSII activity of the three *Chlamydomonas* strains in mid‐exponential growth phase (OD_750_ = 0.6 ± 0.05). The *F*_*v*_ */F*_*m*_ ratio was calculated as *F*_*v*_ */F*_*m*_ = (*F*_*m*_−*F*_*0*_)/*F*_*m*_ and a proxy for the PSII ET efficiency as *1−V*_*J*_ = 1−(*F*_*2ms*_−*F*_*0*_)/(*F*_*m*_−*F*_*0*_), according to (Strasser et al., 2000). Average values of at least three independent experiments are presented ± SE (pigment content, *n* = 9; fluorescence parameters, n = 9–12).

Strains	Chl (*a + b*)	Carotenoids	Chl *a*/*b*	*F*_*v*_ */F*_*m*_	*1−V*_*J*_
	μg ml^−1^	μg ml^−1^	ratio		
**IL**	9.4 ± 2.0	2.1 ± 0.4	2.2 ± 0.00	0.77 ± 0.006	0.53 ± 0.011
**F265T**	4.5 ± 1.0	1.2 ± 0.3	2.4 ± 0.01	0.65 ± 0.010	0.21 ± 0.010
**F265S**	3.9 ± 0.4	1.2 ± 0.2	2.5 ± 0.01	0.49 ± 0.009	0.11 ± 0.005

#### 3.2.2 Light response of oxygen evolution and rETR

**FIGURE 2:**
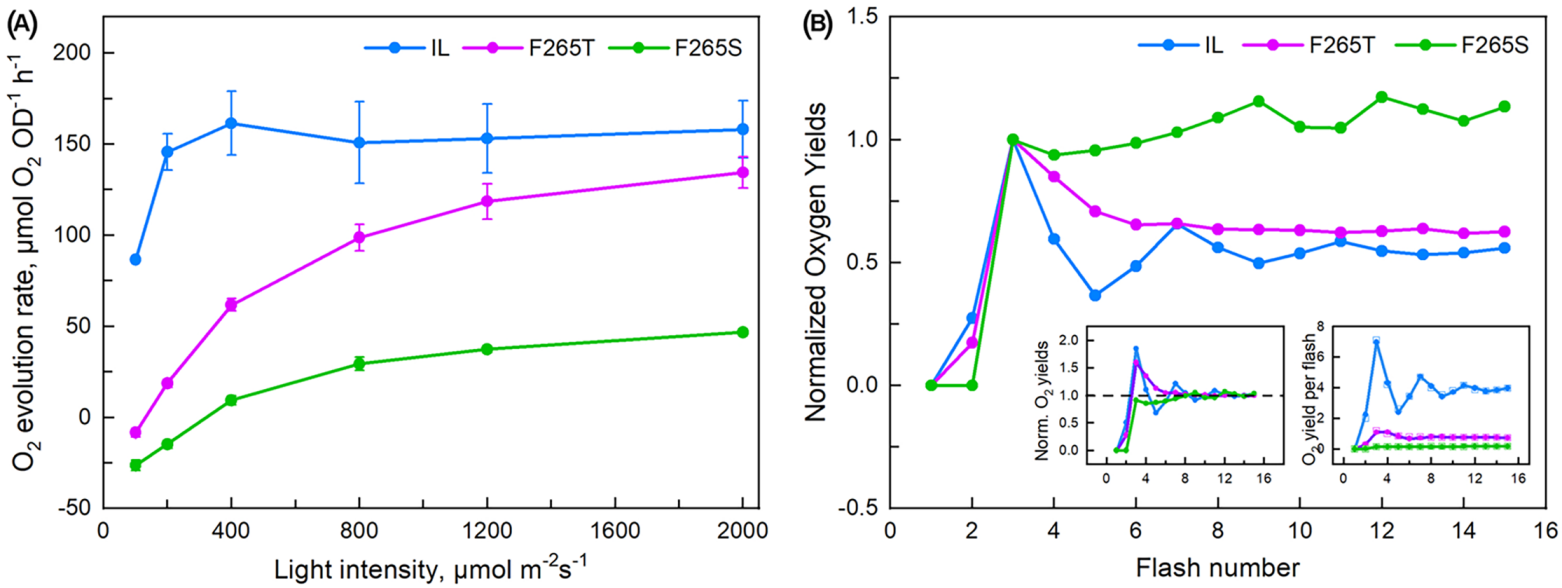
**Oxygen evolution of *Chlamydomonas* strains**. (A) Light response curves of the oxygen production rate of the three *Chlamydomonas* strains registered for 30 s on samples exposed for 30 s at the indicated light intensities. Each point represents an average of three independent experiments with 3 technical repetitions, ± SE (*n* = 3). (B) Oxygen‐flash yields induced by a train of 15 saturating single turnover flashes normalized to the 3rd flash. The inserts present values normalized to the average of the last 5 yields (left insert) and experimental (squares) and theoretical (according to the model of (Kok et al., 1970), circles) values (right insert). Each measurement represents an average of two independent experiments with 2 or 3 technical repetitions, *n* = 4–6.

The photosynthetic performance of the mutants was investigated by measuring photosynthetic oxygen evolution under continuous illumination, in the absence of exogenous electron acceptors (Figure 2A). The light response of oxygen evolution was recorded in the 100–2000 μmol m^−2^ s^−1^ PPFD range. In IL, oxygen evolution was saturated at PPFD 400 μmol m^−2^ s^−1^ and in F265T and F265S, photosynthetic oxygen production prevailed over respiration only at PPFD higher than 100 μmol m^−2^ s^−1^ and 400 μmol m^−2^ s^−1^, respectively. The photosynthetic activity of the mutants was enhanced with light intensity in the 100–2000 μmol m^−2^ s^−1^ PPFD range. This pattern was well pronounced in F265T whose oxygen production at PPFD 2000 μmol m^−2^ s^−1^ was only slightly below the maximum rate of the IL strain. The oxygen evolution rate of F265S was much lower and did not reach one third of the IL rate at PPFD 2000 μmol m^−2^ s^−1^ (Figure 2A).

*rETR* fluorescence measurements showed a similar pattern of differences between the strains as the light response curves of oxygen evolution (Figure S7, Table S2), except that the saturation of *rETR* in IL was reached at a higher light intensity than saturation of oxygen evolution of the same strain (Figure 2A). All strains showed a decrease in *rETR* at light intensities above saturation, a phenomenon that is not linked to known physiological functions (Havurinne et al., 2019).

#### 3.2.3 Flash‐induced oxygen yield

To get further insights into the effects of PSII D1 protein mutations on the functions of the photosynthetic apparatus in *C. reinhardtii* we measured oxygen yields induced by a train of saturating single turnover flashes. Oxygen yields obtained without an added exogenous electron acceptor were very low and therefore phenyl‐*p*‐benzoquinone was added to facilitate the oxidation of Q_B_. Figure 2B shows a normalized (divided by the 3rd flash) pattern of O_2_ flash yields of the reference strain IL and the F265 mutants.

In IL, flash‐induced oxygen evolution has a maximum on the third flash and shows a four oscillation period, characteristic of an active water splitting system (Kok et al., 1970), and the distribution of dark S‐state populations in IL (Table 2) was similar to the one usually obtained for healthy photosynthetic organisms (Kok et al., 1970; Ananyev et al., 2016). The F265T mutant, in turn, showed a low‐amplitude signal (maximum approximately 11% of that of IL). In F265S, flash induced oxygen production was even more severely affected. In both mutants, the third flash gave the maximum oxygen yield, but the further oscillation of the signal was weak in F265T, and in F265S the oscillation was reduced to a slight decrease of oxygen production from the third to the fourth flash, in both mutants the oscillation dampened rapidly (Figure 2B). The oxygen yield of F265S increased slowly toward a second maximum at the 9th flash. Analysis of the results with the Kok model revealed a standard pattern for the IL strain whereas the miss parameter had very high value in both mutants (Table 2). If the dark ratio of S_0_ to S_1_ was let run free, very high S_0_/S_1_ ratios were often obtained for both mutants. However, fitting the data with the S‐state distribution fixed to the standard 25/75 ratio caused only a small increase in the squared error, and the Akaike Information Criterion values for a model with a fixed S_0_/S_1_ ratio (2 parameters) to the free‐running model (3 parameters) were in both cases lower (−11.3 vs. −9.9 for F265T and − 28.0 vs. −26.1 for F265S) for the model with the S_0_/S_1_ ratio fixed to 25/75. Thus, the dark distributions of the S‐states could not be determined for the mutants.

**TABLE 2:** Kinetic parameters of oxygen evolving reactions according to Kok's model (Kok et al., 1970). *S*_*0*_ and *S*_*1*_ represent the distribution of the states of oxygen evolution complex in the dark (n.d. = not determined); *α* and *β* represent the probability of a miss and a double hit, respectively. Each measurement represents an average of two independent experiments with 2 or 3 technical repetitions, ±SE (n = 4–6).

Strain	*S*_*0*_ (%)	*S*_*1*_ (%)	*α*	*β*
IL	27 ± 2	73 ± 2	0.14 ± 0.003	0.12 ± 0.005
F265T	n.d.	n.d	0.27 ± 0.03	0.14 ± 0.05
F265S	n.d	n.d	0.32 ± 0.05	0.13 ± 0.07

#### 3.2.4 Chl *a* fluorescence induction

**FIGURE 3:**
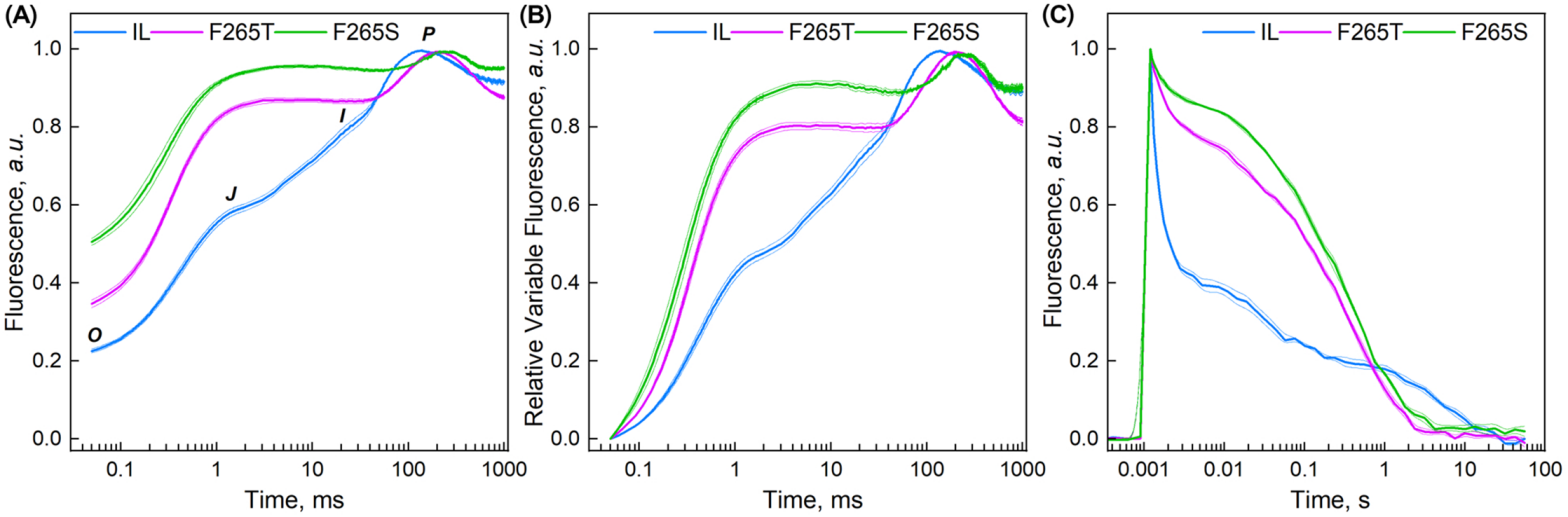
**Chl *a* fluorescence of *Chlamydomonas* strains**. Chl *a* fluorescence transients (A), relative variable fluorescence curves (*V*_*t*_) (B) and fast kinetics of Q _A_^−^ reoxidation after a strong single turnover flash (C) of the three *Chlamydomonas* strains at room temperature. Fluorescence signals were normalized as follows: (A) to the maximum fluorescence, where *F*_*m*_ of IL = 392 ± 30, *F*_*m*_ of F265T = 396 ± 10 and *F*_*m*_ of F265S = 342 ± 21; (B) following the equation *Vt* = (*F−F*_*0*_ *)/(F*_*m*_ *−F*_*0*_); (C) following the equation *F = (F*_*raw*_ *−F*_*0*_ *)/(F*_*150μs*_ *−F*_*0*_). Each curve represents an average of four‐six independent experiments, ± SE (n = 4–6) for (A) and (B) and an average of three independent experiments, ± SE (n = 3) in (C).

In line with the slow cell proliferation under photoautotrophic and mixotrophic conditions, the photochemical reactions of the mutants were also severely altered (Table 1). In comparison with the IL strain, the *F*_*v*_ */F*_*m*_ fluorescence parameter of F265T and F265S was reduced by 16% and 36%, respectively. In F265T, the low *F*_*v*_ */F*_*m*_ was accompanied by 50% higher *F*_*0*_, the minimal fluorescence level, than in IL, while in F265S *F*_*0*_ was twice as high as in IL (Figure 3A).

*OJIP* transients suggested that the mutations slow down ET from Q_A_ to Q_B_, as the *1−V*_*J*_ parameter was reduced by 60% and 80% in F2565T and F265S, respectively, in comparison to the reference strain (Table 1 and Figure 3B). In fact, *OJIP* transients of the two mutants resembled transients measured from WT PSII in the presence of subsaturating concentrations of the herbicide DCMU (Boisvert et al., 2006). Furthermore, the illumination time needed to reach the maximum fluorescence level at the *P* step was longer in the mutants than in the reference strain; 134 ± 7 ms in IL, and 204 ± 6 ms and 236 ± 12 ms in F265T and F265S, respectively, in line with the slow Q_A_‐Q_B_ ET in the mutants.

#### 3.2.5 Relaxation kinetics of flash‐induced Chl *a* fluorescence yield

The effects of D1‐Phe265 substitutions on Q_A_ reoxidation were evaluated by measuring the relaxation kinetics of flash‐induced Chl *a* fluorescence (Figure 3C). In the reference strain IL, the fluorescence decay was dominated by a fast component (66% amplitude and 0.36 ms lifetime) reflecting Q_A_^−^ reoxidation via transfer of an electron on Q_B_ or Q_B_^−^ (Table 3). The middle phase, with 37 ms lifetime and 18% amplitude, reflects Q_A_^−^ reoxidation in PSII without an oxidized or singly reduced PQ molecule in the Q_B_ binding site. The slow phase, assigned to charge recombination of Q_A_ˉ with the S_2_ state of the OEC in PSII centres incapable of reducing Q_B_, represented 16% of the amplitude of fluorescence decay in the IL strain. In the mutants, forward ET was considerably slower than in IL, as demonstrated by the long lifetimes of the fast and the middle components of the decay (Table 3). In agreement with the small relative amplitude of the *J‐P* rise in the *OJIP* curves (Figure 3A), the relaxation of Chl *a* fluorescence yield after a single turnover flash supports the conclusion that forward ET from Q_A_^−^ after a flash is slow in the D1‐Phe265 mutants, as the amplitude of the slow phase of the relaxation, interpreted as S_2_Q_A_^−^ → S_1_Q_A_ charge recombination, comprised approximately half of the decay in both mutants (Figure 3c, Table 3).

**TABLE 3:** Relative amplitudes (*A*) and lifetimes (*τ*) of the components of decay of Chl *a* fluorescence yield after a single turnover flash in the *Chlamydomonas* strains. The analysis is based on the data in Figure 3C.

Strains	Forward ET				Charge recombination	
	*A*_*fast*_ (%)	*τ*_*fast*_ (ms)	*A*_*middle*_ (%)	*τ*_*middle*_ (ms)	*A*_*slow*_ (%)	*τ*_*slow*_ (s)
**IL**	66	0.36	18	37	16	6.3
**F265T**	24	0.75	23	56	53	0.7
**F265S**	15	0.69	36	100	49	1.0

The lifetime of the slow component (≈1 s in the mutants *vs* ≈6 s in IL) might suggest that the activation energy of S_2_Q_A_^−^ charge recombination (Rappaport et al., 2005; Perrine & Sayre, 2011) is lower in the mutants than in IL. However, measurements of the decay of fluorescence yield in the presence of DCMU, when the recombination of the S_2_Q_A_^−^ state is the only reoxidation pathway for Q_A_^−^, showed recombination with roughly 1 s kinetics in all strains (Figure S8, Table S3). This reaction could be analysed in our conditions with a minor fast (amplitude 36–43%, lifetime 100–115 ms) and a major slow exponential phase (amplitude 57–64%, lifetime 0.96–1.01 s) at 25°C in all three *Chlamydomonas* strains (Table S3). The temperature dependences of both phases showed Arrhenius‐type behaviour and revealed that the activation energy of the fast phase was lower in both mutants than in IL, whereas that of the major slow phase was similar in F265T and IL and higher in F265S than in IL (Table S4).

#### 3.2.6 Thermoluminescence (TL) measurements

**FIGURE 4:**
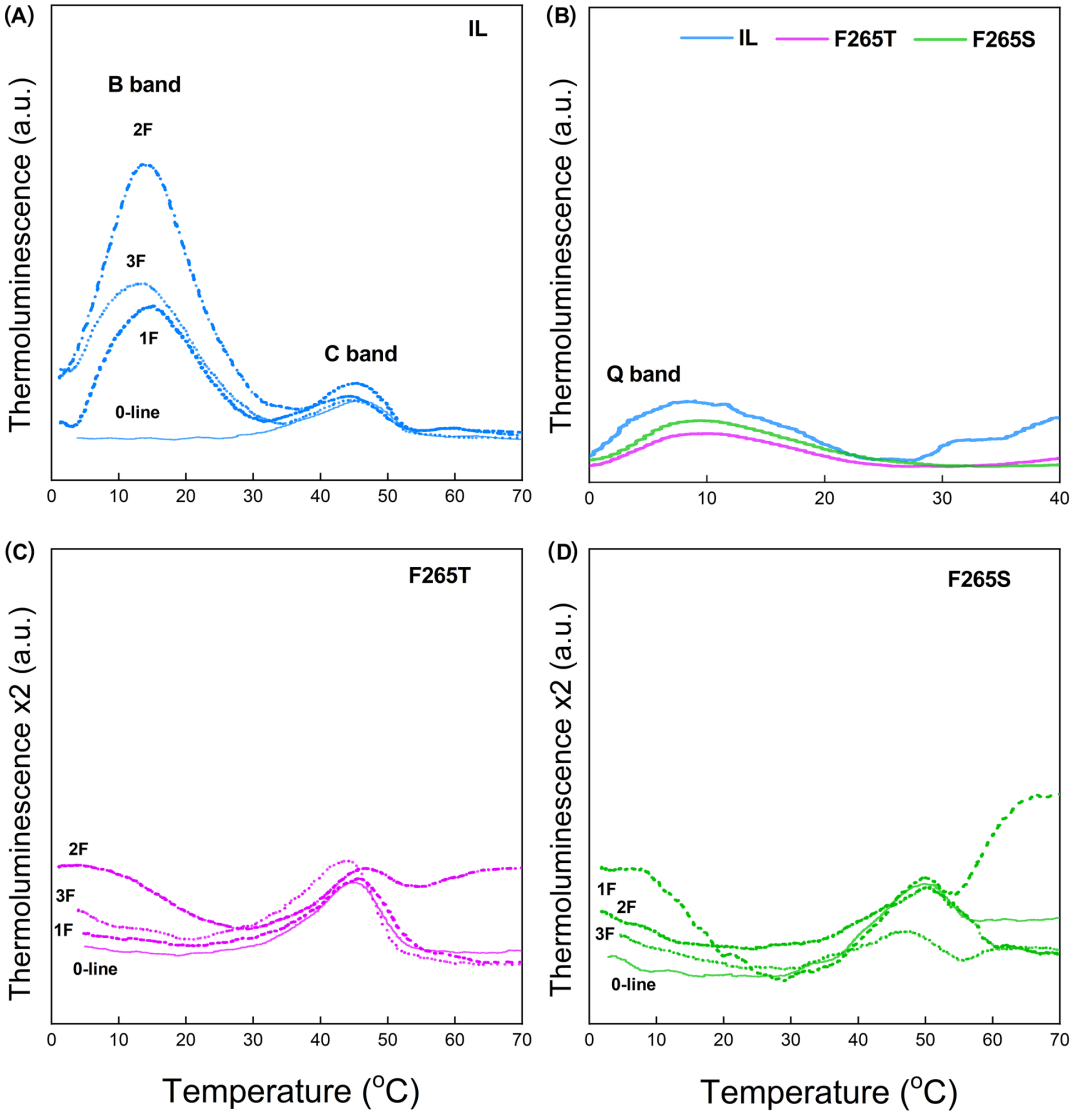
**TL emission curves of the three *Chlamydomonas* strains recorded at 0.5 °C s** ^**−1**^ **heating rate**. TL in IL (A), IL, F265T and F265S in the presence of 10 μM DCMU, 1F (B), F265T (C) and F265S (D), where 1F, 2F and 3F indicated excitation by one, two or three saturating single turn‐over xenon flashes respectively. 0‐line corresponds to the TL signal measured on samples not subjected to any flashes. Representative curves are presented, *n* = 5.

TL was used to probe the effects of the D1‐Phe265 substitution on charge recombination between the donor and acceptor sides of PSII. The measurements were done on intact cells without freezing the samples (Ducruet et al., 2011; Repetto et al., 2015). The B luminescence band, obtained without adding DCMU, peaked at 15–20°C in the IL strain, and the highest B‐band was registered after two flashes (Figure 4A). The B band originates from the recombination reactions S_2_Q_B_^−^ → S_1_Q_B_ and S_3_Q_B_^−^ → S_2_Q_B_, where S_2_ and S_3_ are states of the OEC and Q_B_ is the secondary quinone acceptor of PSII (Rutherford et al., 1984). The B band of *C. reinhardtii* peaks at a lower temperature than that of plants (Ducruet et al., 2007; Virtanen et al., 2021).

When ET to Q_B_ is blocked by DCMU, the so obtained Q band peaks at a lower temperature than the B band because only S_2_Q_A_^−^ → S_1_Q_A_ and S_3_Q_A_^−^ → S_2_Q_A_ recombination occur (Rutherford et al., 1982; Tyystjärvi & Vass, 2004). In F265T and F265S, the Q band peaked at the same temperature as in IL (Figure 4b).

TL curves measured in the absence of DCMU from the two mutants (Figure 4C, D) indicated a maximum at a lower temperature than that obtained in the presence of DCMU (Figure 4B). The oscillation pattern of F265T resembled that of IL, indicating that some advancement of the S‐states occurs in this mutant (Figure 4C). In the TL emission curves of all strains an additional small band was visible in the range of 45–50°C: it appeared in absence of a flash (0 ‐ line) and after one to three flashes (1F, 2F and 3F). In F265S, oscillation by flash number occurred but the pattern was abnormal, as the highest intensity was obtained after one flash (Figure 4D).

### 3.3 Oxygen evolution and growth of *Synechocystis* strains PD and AR

The PD mutant (Δ225‐239 of *psbA2* gene) of the cyanobacterium *Synechocystis* sp. PCC 6803 has earlier been shown to have slow decay of chlorophyll *a* fluorescence yield after a single‐turnover flash (Mulo et al. 1997) and overlapping Q and B bands (Keränen et al. 1998), thus resembling the F265T and F265S mutants of *C. reinhardtii* in these respects. The DPEST mutant (Δ226‐233 of *psbA3* gene) shows similar properties (Nixon et al. 1995). The large deletion of PD is very close to the bicarbonate ligand D1‐Y246 (Nihara et al. 2025) and thus probably hampers electron transfer through an effect on bicarbonate binding.

**FIGURE 5:**
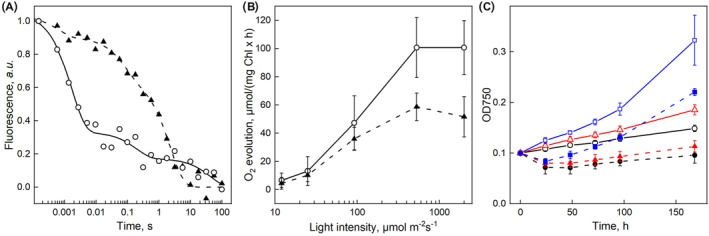
**Experiments with the PD strain of *Synechocystis* sp. PCC 6803**. (A) Decay of Chl *a* fluorescence yield after a single turnover flash in the AR control strain (circles) and the PD strain (triangles). The lines (solid line, AR; dashed line, PD) show the best fit to the sum of three exponential components. (B) Light response of photosynthetic oxygen evolution (H_2_O to dichlorobenzoquinone) in the AR control strain (circles) and the PD strain (triangles). (C) Growth of AR (open symbols, solid lines) and PD (solid symbols, dashed lines) at 6 (circles), 12 (triangles) and 20 μmol m^−**2**^ s^−**1**^ (squares). Growth was followed in 30 mL volume at 32°C and 1% CO_2_. Each symbol represents an average of three independent experiments and the error bars in (B) and (C), drawn larger than the symbol, show SD.

Earlier experience with the PD strain has shown that the mutant tends to revert to the wild type, and therefore DCMU was included, in addition to antibiotics, for growing the *Synechocystis* strains on plates. Furthermore, the decay of Chl *a* fluorescence yield after a single turnover flash was measured to ensure that no reversion had occurred (Figure 5A). Contrary to the F265S and F265T *Chlamydomonas* strains, the rate of oxygen evolution of the PD strain was nearly similar as in the control strain AR in low light, but the mutant lagged behind in strong light (Figure 5B). Furthermore, the PD strain grew at very low PPFD values at similar rates as the AR strain after a cell density drop of unknown origin occurring in PD at the beginning of each growth experiment (Figure C).

### 3.4 Modelling of the light response of photosynthesis

The F265T and F265S *Chlamydomonas* mutants share with the PD strain of *Synechocystis* sp. PCC 6803 an overlap of the TL bands Q and B, and slow decay of Chl *a* fluorescence yield after a single turnover flash, indicating that Q_A_‐Q_B_ electron transfer is slow in all three mutants. However, the light response curve of PD differs sharply from that of the *Chlamydomonas* mutants. To get insight to the difference, we modelled PSII electron transfer, using a rate constant for the fastest phase of Q_A_‐Q_B_ electron transfer and for charge recombination similar to those measured for the IL strain (Table 3). The rate constant for electron transfer from Q_A_^−^ to Q_B_^−^ was set to 300 s^−1^ and the rate constants for binding of a PQ molecule to an empty Q_B_ site and for the release of PQH_2_ were set to 50 s^−1^. The latter three rate constants affect the maximum rate of electron transfer. Both equilibria of electron sharing between Q_A_ˉ and Q_B_^−^/Q_B_^2^^−^ were set to 1/20 (backward rate constants 138 and 15 s^−1^). Then, a mutation zeroing the driving force for Q_A_‐Q_B_ electron transfer was modelled by setting both equilibria to 1 (backward rate constants 2777 and 300 s^−1^), and the effect of a mutation slowing down the protonation of Q_B_^2^ˉ was modelled by setting the rate constant for the release of PQH_2_ to 6.25 s^−1^ (1/8 of the wild‐type value) while keeping all other rate constants in the wild‐type values. The Copasi software (Hoops et al. 2006) was used for the modelling.

**FIGURE 6:**
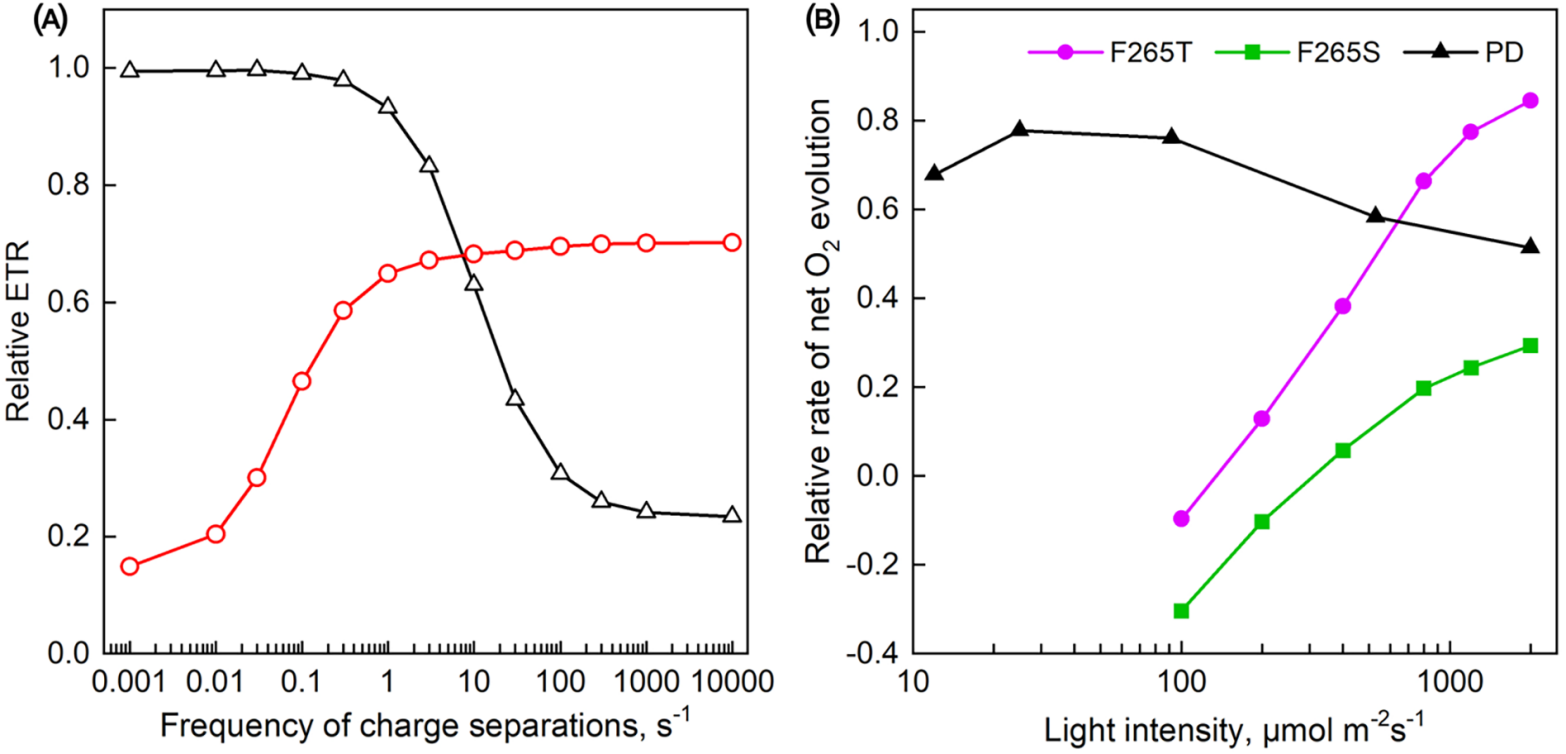
**Modelling the effects of mutants on the light response of PSII electron transfer**. (A) The effect of a mutation zeroing the driving force of Q_A_‐Q_B_ electron transfer (red circles) and a mutation that slows the protonation of Q_B_^2−^ to 1/8th of the wild‐type value (black triangles). Relative ETR is the ETR in a mutant divided by ETR in wild type. See Materials and Methods for the specifications of the model. (B) Relative rate of net O_2_ evolution (rate in mutant divided by rate in wild type) in F265T (magenta circles) and F265S (green squares) mutants of *C. reinhardtii* and in the PD mutant of *Synechocystis* sp. PCC 6803 (black triangles).

The results of the modelling show that the lack of the driving force for Q_A_‐Q_B_ electron transfer would slow down ETR predominantly in low light whereas slow protonation of Q_B_^2^^−^ would slow down ETR predominantly in strong light (Figure 6A). Experimental data from the *C. reinhardtii* mutants F265T and F265S clearly resemble the model data for a mutant with no driving force (Figure 6B). The PD mutant of *Synechocystis* sp. PCC 6803, in turn, shows a 34% decrease in the ratio of O_2_ evolution rate to the respective wild‐type rate from the maximum reached at PPFD 25 μmol m^−2^ s^−1^ to the minimum obtained at PPFD 2000 μmol m^−2^ s^−1^ (Figure 6B).

### 3.5 Affinity of Chlamydomonas strains to ATZ

**FIGURE 7:**
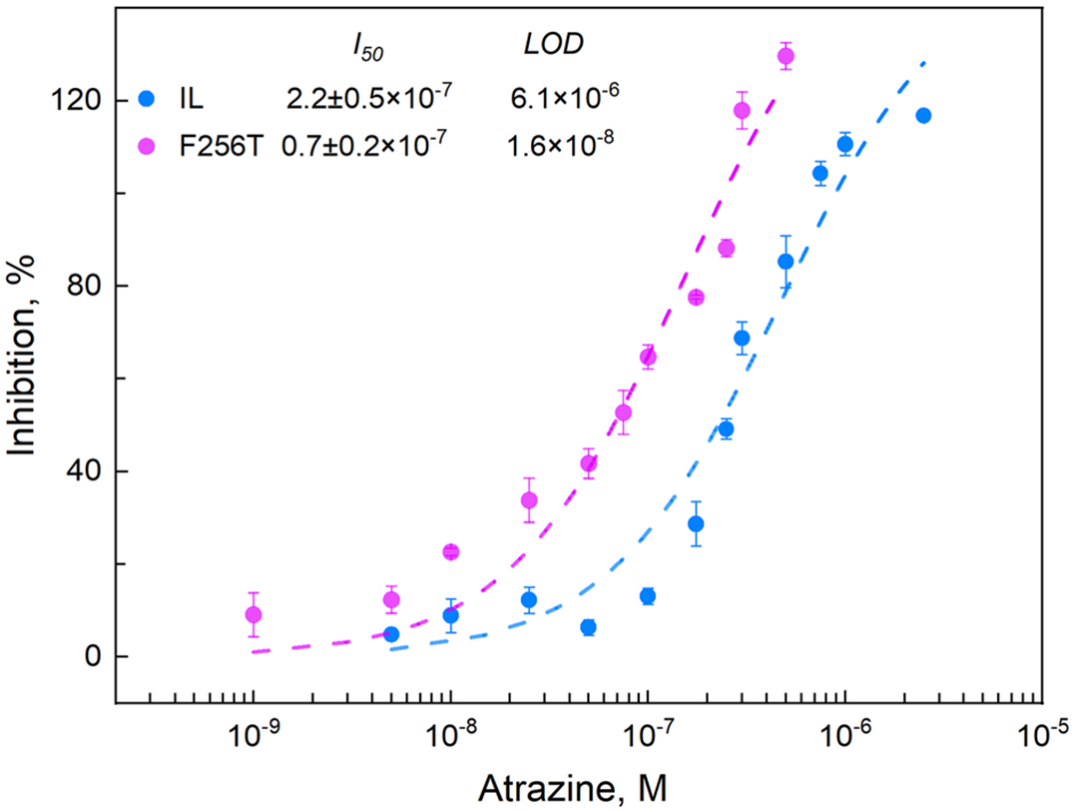
**Dose–response curves of oxygen evolution rate to ATZ measured on the reference strain IL and F265T *Chlamydomonas* mutant**. Average values of three to seven independent experiments for each herbicide concentration are presented, ± SE (n = 3–7).

Measurements of Chl *a* fluorescence yield after a single turnover flash (Figure S9) show that an enhanced ATZ sensitivity would be difficult to detect from the F265S and F265T mutants also using a different type of Chl *a* fluorescence measurement. These considerations led us to assess the binding affinity of the mutants in terms of oxygen evolution rate which is, in our opinion, a trustable parameter for the development of electrochemical biosensors. The capability of the F265T (and IL) strains to detect ATZ through dose–response curves of oxygen evolution in the presence of 1 × 10^−9^ – 1 × 10^−6^ M ATZ (Figure 7), indicated *I*_*50*_ and LOD values 3.2 and 3.8 times lower, respectively, for D1‐F265T than for the reference strain IL. Assuming that the *I*_*50*_ parameter is equivalent to the *K*_*D*_ dissociation constant (Shitanda et al., 2009), this result verified the in silico prediction that the Q_B_ binding niche of the F265T mutant has a higher affinity for ATZ than the native Q_B_ binding pocket. The achieved LOD value of 1.6 × 10^−8^ M (corresponding to 3.45 μg l^−1^) is close to the Maximum Residue Limit (MRL) for single pesticide in drinking waters established by the United States Environmental Protection Agency (3 μg l^−1^), while it is far from the MRL set by the EU Council Directive 98/83/EC (US&EPA) (0.1 μg l^−1^).

## 4 DISCUSSION

### 4.1 Molecular docking and MD simulations

Previous research has demonstrated that specific mutations at the residues within the Q_B_ binding site of the D1 protein can lead to alterations in stereochemical, electrostatic, and hydrophobic interactions. These changes can result in conformational shifts, reorganization of hydrogen bond networks, and variations in protein dynamics, ultimately affecting the binding of herbicides (Lambreva et al., 2014; Zobnina et al., 2017; Brown et al., 2024).

Molecular docking and MD simulations uncovered the precise interactions between natural PQ or ATZ and amino acid residues within the Q_B_ niche and suggested that substituting the D1‐F265 residue with a polar hydrogen bond acceptor residue could stabilize the atrazine N5 proton and ATZ binding within the pocket, resulting in increased affinity. These predictions were validated by examining the binding affinity of ATZ in the newly produced D1‐F265T mutant of *C. reinhardtii* through dose–response curve experiments, that highlighted the critical importance of even a single amino acid substitution in the Q_B_ binding pocket for maintaining the physicochemical properties and functionality of the D1 protein. A similar conclusion was reached by comparing the functional dynamics – the relationship between atomic and molecular motions and their effects on biomolecular activity – of the IL and F265T thylakoid membranes. Neutron scattering experiments indicated that the enhanced flexibility of the F265T mutant at the nanosecond timescale is associated with a less functional system. This observation is consistent with findings in *Rhodobacter spheroides*, which has mutated the L protein and demonstrates impaired electron transport (Russo et al., 2019). Furthermore, the F265 mutation results in a shift of the Q_B_/Q_B_^−^ redox potential to a more negative value, which correlates with enhanced ATZ sensitivity. This finding does not align with previous data pointing out to an inverse relationship observed in D1 mutants hosting mutations in close proximity to the F265 residue in the primary structure, e.g., S264. This observation leads to the conclusion that the impacts of single amino acid substitutions on ATZ binding affinity at the Q_B_ site can vary significantly, even among residues that are close to each other in the primary structure. This variability underscores the importance of considering the spatial arrangement of these residues in three‐dimensional space. Additionally, the chemical nature of the substituted amino acid plays a critical role in shaping the molecular environment – affecting structure, dynamics, and physicochemical properties – thereby influencing binding specificity and strength. Hence, the impact of mutations at the D1‐F265 residue on atrazine binding affinity to the Q_B_ site is distinctive, despite a comparable shift in the Q_B_/Q_B_^−^ redox potential was observed in other nearby mutations conferring atrazine resistance.

The potential of the F265T mutant as a biosensing element is promising, although the achieved LOD values, tested in algal cultures rather than in a complete biosensor device, exceed international standards. Biosensors combine biological components with chemistry and electronics, and their sensitivity can be improved by using nanomaterials, which offer signal amplification, high surface area, and compatibility with various sensing platforms. These enhancements can significantly lower LOD values, enabling the detection of trace herbicide amounts in environmental and agricultural contexts. Additionally, improving transducer designs and integrating concentration systems with biosensors can further boost sensitivity.

### 4.2 Chl *a* fluorescence reveals that forward ET from Q_A_ is slow in F265T and F265S

The F265T and F265S strains showed a low *F*_*v*_ */F*_*m*_ that has been shown to correlate with a low rate of ET through PSII in photoinhibition (Tyystjärvi, 2013), in heat damage to PSII (Pospíšil & Tyystjärvi, 1999) and in various PSII mutants (Clarke et al., 1993; Rea et al., 2011b; Lambreva et al., 2013; Cecchin et al., 2021). Thus, the data suggest that PSII of both mutants functions inefficiently although the high‐fluorescence *F*_*m*_ state is actually formed partly due to conformational changes induced by exposure of a closed PSII to light (Sipka et al., 2021). In both mutants, low *F*_*v*_ */F*_*m*_ was caused by a high value of *F*_*0*_, suggesting that some fraction of PSII centres remained closed throughout the dark incubation. PSII centres can stay closed in the dark if the redox equilibrium between Q_A_/Q_A_ˉ and PQ/PQH_2_ does not favour forward ET (Tsimilli‐Michael, 2020). The equilibrium, in turn, depends on the redox potential difference of the Q_A_/Q_A_ˉ and Q_B_/Q_B_^−^ pairs and the availability of oxidized PQ molecules in the PQ pool. The results of the *OJIP* curves of the two mutants showed a high *J* level and delay in reaching the *P* level, in agreement with the conclusion of slow forward ET from Q_A_^−^ (Figure 3A,B). Furthermore, the decay of fluorescence yield after the *P*‐peak was slow in both mutants (Figure 3C), indicating that photochemical and non‐photochemical quenching build up slowly, obviously because ET through PSII is slow.

The decay of Chl *a* fluorescence yield after a single turnover flash reflects Q_A_^−^ reoxidation (Crofts et al., 1993; Tyystjärvi & Vass, 2004). Results obtained from both mutant strains in the absence of the herbicide DCMU (Figure 3C) resemble data obtained when Q_A_^−^ re‐oxidation is inhibited by binding DCMU to the Q_B_ site (Figure S8; Antal & Rubin, 2008; Lambreva et al., 2013). In the presence of DCMU, in turn, the data show that the recombination of the S_2_Q_A_^−^ state was not strongly affected by the mutations (Figure S8). The low activation energy of both components of fluorescence decay in F265T was compensated by a lower preexponential factor (Table S4). The decay of fluorescence yield in the presence of DCMU did not show any sign of a very fast component that would indicate malfunction of the OEC (Boerner et al., 1992; Allahverdiyeva et al., 2004; Antal et al., 2013).

In the PD strain of the cyanobacterium *Synechocystis* sp. PCC 6803, the decay of Chl *a* fluorescence yield (Figure 5A) was highly similar as in the F265T and F265S *Chlamydomonas* strains (Figure 3C).

### 4.3 F265T and F265S lack a driving force for Q_A_ to Q_B_ ET

In addition to a small or missing redox potential difference between the Q_A_/Q_A_^−^ and Q_B_/Q_B_^−^ pairs, other reasons can slow down the rate of Q_A_‐Q_B_ electron transfer, including slow exchange of PQH_2_ to PQ in the Q_B_ site or a permanently highly reduced state of the plastoquinone pool. Mutations in D1‐F265 might disturb the hydrogen‐bond network that protonates Q_B_^−^ or Q_B_^2^^−^ (Shevela et al., 2012; Forsman and Eaton‐Rye 2020). We cannot completely rule out an effect of the mutations on the exchange of PQH_2_ to PQ in the Q_B_ site, especially as D1‐Phe265 has been suggested to stabilize Q_B_ binding (Cardona et al., 2012; Zobnina et al., 2017; Kulik et al., 2020), but we will show in the next paragraphs that the main reason for the slow ETR in the mutants is a zero or almost zero redox potential difference between Q_A_/Q_A_^−^ and Q_B_/Q_B_^−^ pairs.

The Q band TL peak, measured in the presence of DCMU, mainly reflects the redox potential of the Q_A_/Q_A_^−^ pair (Krieger‐Liszkay & Rutherford, 1998). The results reveal a similar Q band peak in all three strains, suggesting that the redox potential of the Q_A_/Q_A_^−^ pair is similar in IL, F265T and F265S. This finding is in line with similar temperature dependency of Q_A_^−^ reoxidation kinetics measured in presence of DCMU (Table S3) and with the decay of Chl *a* fluorescence yield after a single turnover flash (Figure 3C), which suggests that ET from Q_A_^−^ to Q_B_ in F265T and F265S is so slow that S_2_Q_A_ˉ charge recombination dominates the kinetics of the decay of Chl *a* fluorescence yield after a single turnover flash (Table 3). The result suggests that the redox potential of the Q_B_/Q_B_ˉ pair is essentially the same as that of the Q_A_/Q_A_ˉ pair in the two mutants (Endo et al., 2015), and therefore there is no driving force for ET from Q_A_ to Q_B_.

Comparison of the effects of the mutations F265T and F265S on the light response of O_2_ evolution with a model of the effects of mutations affecting the driving force and mutations affecting the rate of exchange of Q_B_H_2_ to oxidized PQ in the Q_B_ site (Figure 6) shows that both mutants behave as expected if the driving force is zero, and are in sharp contrast to the modelled behaviour of a mutant with slow exchange reaction. However, an additional effect, e.g., caused by slow protonation of Q_B_^2^^−^, cannot be excluded, especially in the F265S mutant.

The PD mutant strain of *Synechocystis* sp. PCC 6803 offers an example of the opposite behaviour. In this strain, the B and Q TL bands are essentially on top of each other (Keränen et al. 1998) and the decay of Chl *a* fluorescence yield after a single turnover flash resembles data obtained in the presence of DCMU from wild‐type PSII (Figure 5). The PD strain behaves like a mutant in which the exchange reaction is slow, suggesting that in this mutant, not the driving force for Q_A_‐Q_B_ electron transfer but instead the exchange of reduced to oxidized PQ in the Q_B_ site is disturbed (Figure 6). For the PD mutant, slow exchange is expected, as the mutation is a large deletion near to the binding site of the bicarbonate ion of PSII. The finding suggests reconsideration of earlier conclusions about energetic destabilization of the Q_B_ quinone due to deletions of PEST‐like sequence of the D1 protein (Nixon et al., 1995; Mulo et al., 1997).

A further consequence of the slow Q_A_‐Q_B_ electron transfer is that the actual B band that would reflect recombination of Q_B_^−^ with the S_2_ or S_3_ state of the OEC, is weak in the F265T and F265S *Chlamydomonas* mutants. In fact, if the Q_A_/Q_A_^−^ and Q_B_/Q_B_^−^ pairs have the same redox potential, then the B band reflects recombination from the Q_A_^−^Q_B_↔Q_A_Q_B_^−^ equilibrium. The oscillation of the intensity of the B band in both F265T and F265S (Figure 4), even if the band position is far below that expected for the B band, supports this interpretation.

The TL band obtained in the absence of DCMU from F265S or F265T peaked at a lower temperature than the Q band obtained in the presence of DCMU (Figure 4). The most likely reason for this is a DCMU‐induced shift in the redox potential of the Q_A_/Q_A_^−^ pair to a more positive value (Krieger‐Liszkay & Rutherford, 1998; Fufezan et al., 2002). In support of this hypothesis, it has earlier been shown that a large deletion in the DE loop of the D1 protein of the cyanobacterium *Synechocystis* sp. PCC 6803 (PCD mutant) leads to slow decay of Chl *a* fluorescence yield after a single turnover flash (Mulo et al., 1997) and to a shift of the B band to a lower temperature than the Q band of the same mutant (Keränen et al., 1998).

The secondary peak, the so‐called C band, observed in all strains (Figure 4), reflects TyrD^+^Q_A_ˉ recombination in PSII centres lacking a functional OEC, and the band is fairly stable in the dark (Johnson et al., 1994). The band has a similar amplitude in IL and in the mutants, suggesting that the mutations do not seriously affect the function of the OEC.

### 4.4 Driving force for Q_A_ ‐Q_B_ ET matters in weak light but not in strong light

The flash oxygen yield patterns of the mutants are in good agreement with ET kinetics. In wild‐type PSII, conversion from S_n_Q_A_^−^Q_B_^(^^−^^)^ to S_n + 1_Q_A_Q_B_^(^^−^^)^^−^ occurs within the 300 ms delay between the flashes in 84% of PSII (Table 3) but in F265T and F265S, the equilibrium constant of the Q_A_ˉQ_B_↔Q_A_Q_B_ˉ reaction is nearly unity, as indicated by the finding that the percentage of apparently slowly reacting PSII centres is 53 and 49% of PSII in F265T and F265S mutant, respectively (Table 3). Assuming that the fraction of closed PSII centres is equal to the normalized yield of variable fluorescence at any time, then the data in Table 3 suggest that if flashes are fired at 300 ms intervals, the n^th^ flash encounters a fraction of 0.847^(n−1)^, 0.654^(n−1)^ or 0.619^(n−1)^ open PSII reaction centres in IL, F265T and F265S, respectively. This translates to a high miss parameter in the flash oxygen data (Table 2). Thus, the two mutants show that a drastic alteration of the PSII acceptor side can produce a high miss factor when misses are analysed with oxygen flash sequences, although the misses in wild‐type PSII originate on the oxidizing side (Pham et al., 2019; Mattila et al., 2022). Strong dampening of the flash number dependence of the oxygen yield and a corresponding high miss factor have been measured from *Synechococcus* PCC7942 strains with single‐site mutation in *psb*A1 at position 264 of D1 (Gleiter et al., 1992). A qualitatively similar effect can be obtained by herbicides that block ET from Q_A_ to Q_B_ (Yotsova et al., 2017).

The fluorescence (Figure 3) and TL data (Figure 4) indicate that the equilibria of the reactions Q_A_^−^Q_B_^(^^−^^)^_↔_ Q_A_Q_B_^(^^−^^)^^−^ are near to unity in the two mutants. This finding seems to be at odds with the relatively high rates of oxygen evolution measured in high light in these two mutants (especially in F265T). However, data in Figures 3 and 4 reflect the equilibration of the above reaction(s) whereas continuous illumination (Figure 2A; see also Figure 6) maintains a high concentration on the left side, thereby compensating, by a concentration difference, for the lack of a potential difference between Q_A_/Q_A_^−^ and Q_B_/Q_B_^−^. This leads to a net forward reaction according to the law of mass action.

Oxygen evolution of both F265T and F265S increases with light much more slowly than that of IL (Figure 2A). The *rETR* data show a similar trend although saturation of *rETR* in IL required much stronger light than the saturation of oxygen evolution (Figure S7, Table S2, Figure 2A). Although the F265T and F265S mutants are not full models of an ancient homodimeric PSII, the sluggish light response of the two mutants suggests that during the evolution of PSII, a homodimeric type II reaction centre would have required strong light. Thus, a redox potential difference, probably developing through a shift of the Q_A_/Q_A_ˉ pair toward a more negative potential, may be an adaptation to weak light. Thus, our data experimentally verified the suggestion that early homodimeric Type II centres would function but would not be optimal for low light (Cardona et al., 2012).

## AUTHOR CONTRIBUTIONS

M.D.L. wrote the original draft and was involved in further writing at the reviewing and editing phase, conceptualization of the study, formal analysis, investigation, choice of methods, validation and visualization of the data. V.Z. took part in investigation, formal analysis, visualization and writing at the reviewing and editing phase. T.KA. was involved in investigation and formal analysis. V.N.P. was involved in investigation, did formal analysis of the data and was involved in writing the original draft, visualization and writing at the reviewing and editing phase. M.T.G. was involved in the conceptualization. I.B. and U.J. were involved in the investigation. O.V. and M.R. were involved in investigation, formal analysis and visualization. P.M. was involved in formal analysis and provided resources. F.P., E.T. and G.R. were involved in conceptualization, writing the original draft and in further writing at the reviewing and editing phase, formal analysis, methodology, validation, and supervision; they also provided resources.

## FUNDING INFORMATION

This work was supported by grants from European Cooperation in Science and Technology (COST Action TD1102), Regione Lazio (grant n. 85–2017‐15256), CNR project FOE‐2021 DBA.AD005.225, Academy of Finland (grant n. 333421) and by Novo Nordisk Fonden (grant NNF220C0079284).

## CONFLICT OF INTEREST STATEMENT

The authors declare that they have no known competing financial interests or personal relationships that could have appeared to influence the work reported in this paper.

## Supporting information


**Appendix S1:** Supporting Information.

## Data Availability

Original data are available in Mendeley Data (DOI: 10.17632/4gy4nthgrd.1).
