# Supplementary material for: Redesign of the Chlamydomonas reinhardtii QB binding niche reveals photosynthesis works in the absence of a driving force for QA ‐QB electron transfer
Source: Physiol Plant. 2024 Dec 14;176(6):e70008. doi: 10.1111/ppl.70008 (PMC11645544; doi:10.1111/ppl.70008)
Supplement: Supplementary file 1 — Appendix S1: Supporting Information. [file PPL-176-e70008-s001.pdf]

Redesign of the *Chlamydomonas reinhardtii* Q_B binding niche reveals photosynthesis works in the absence of a driving force for Q_A-Q_B electron transfer

Maya D. Lambreva[a], Veranika Zobnina[b], Taras K. Antal[c], Violeta N. Peeva[d], Maria Teresa Giardi[e,f], Ivo Bertalan[g], Udo Johanningmeier[g], Olli Virtanen[h,i], Mithila Ray[h], Paula Mulo[h], Fabio Polticelli[b,l]*, Esa Tyystjärvi[h]*, Giuseppina Rea[f]*

[a]Institute for Biological Systems, National Research Council, Via Salaria Km 29.300, 00015 Monterotondo Stazione (RM) Italy; [b]Department of Sciences, University Roma Tre, Viale Guglielmo Marconi 446, 00146 Rome, Italy; [c]Laboratory of integrated ecological research, Pskov State University, Krasnoarmeyskaya st. 1, Pskov 180000, Russia; [d]Bulgarian Academy of Sciences, Institute of Plant Physiology and Genetics, "Acad. G. Bonchev" Str. bl. 21, 1113 Sofia, Bulgaria; [e]Biosensor Srl, Via Degli Olmetti 44, 00060, Formello, Rome, Italy; [f]Institute of Crystallography, National Research Council, Via Salaria Km 29.300, 00015 Monterotondo Stazione (RM) Italy; [g]Institut für Pflanzenphysiologie, Martin-Luther-Universität Halle-Wittenberg, Weinbergweg 10, 06120 Halle (Saale), Germany; [h]University of Turku, Department of Life Technologies/Molecular Plant Biology, 20014 Turku, Finland; [i]Vrije Universiteit Amsterdam, Department of Physics and Astronomy, De Boelelaan 1081, HV Amsterdam, The Netherlands; [l]National Institute of Nuclear Physics, Roma Tre Section, Via della Vasca Navale 84, 00146 Rome, Italy.

***Corresponding authors**:
Fabio Polticelli; E-mail: polticel@uniroma3.it; Telephone: +390657336362
Esa Tyystjärvi; E-mail: esatyy@utu.fi; Telephone: +358405113503
Giuseppina Rea; E-mail: giuseppina.rea@cnr.it; Telephone: +390690672631

Funding
This work was supported by grants from European Cooperation in Science and Technology (COST Action TD1102), Regione Lazio (grant n. 85-2017-15256), CNR project FOE-2021 DBA.AD005.225, Academy of Finland (grant n. 333421) and by Novo Nordisk Fonden (grant NNF220C0079284).

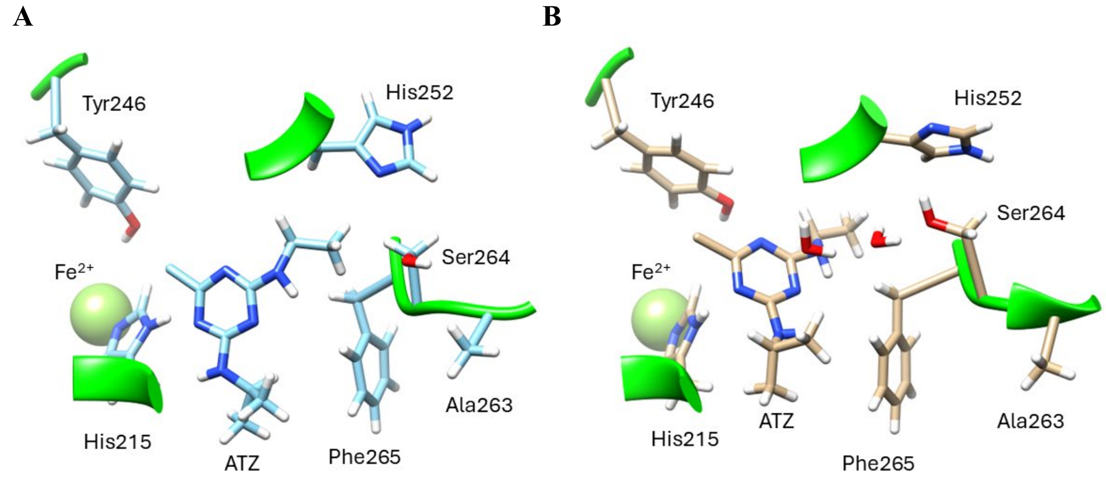

Fig. S1. Snapshots of the conformations of atrazine (ATZ) within the Q_B binding pocket. (A) conformation after energy minimization, (B) conformation after 10 ns MD simulations. During this procedure the ATZ molecule moved towards the helix TMH-d and the non-heme iron atom (towards the core of RC) so that the distance between the centre of mass of the triazine ring and the iron atom was 8.8 Å. The ring nitrogen atom (N1) and the Nδ atom of His215 were within hydrogen bond distance. The distances between the nitrogen (N4) atom of the ethylamino group and the backbone atoms of Phe265 were too long to allow the formation of hydrogen bonds (4.7 Å to oxygen, 4.4 Å to nitrogen). Nonetheless, the interactions of ATZ with the loop region were preserved through water molecules. (N4) and (N5) nitrogen atoms of the ATZ molecule were connected through a double water bridge to the carbonyl oxygen of Ala263. Residues Gly253, Ala263 and Gly256 were no longer within 4.0 Å distance to any atom of the molecule, and a new residue, Leu218 appeared within 4.0 Å distance, close to the Cl atom of the ATZ molecule.

Fig. S2. Schematic representation of PCR-based site-directed mutagenesis used for the production of the D1 mutants (Dauvillee *et al.*, 2004; Rea *et al.*, 2011). The yellow colour indicates the codon encoding for phenylalanine 265 in the native D1 protein; the red colour indicates the newly introduced codons.

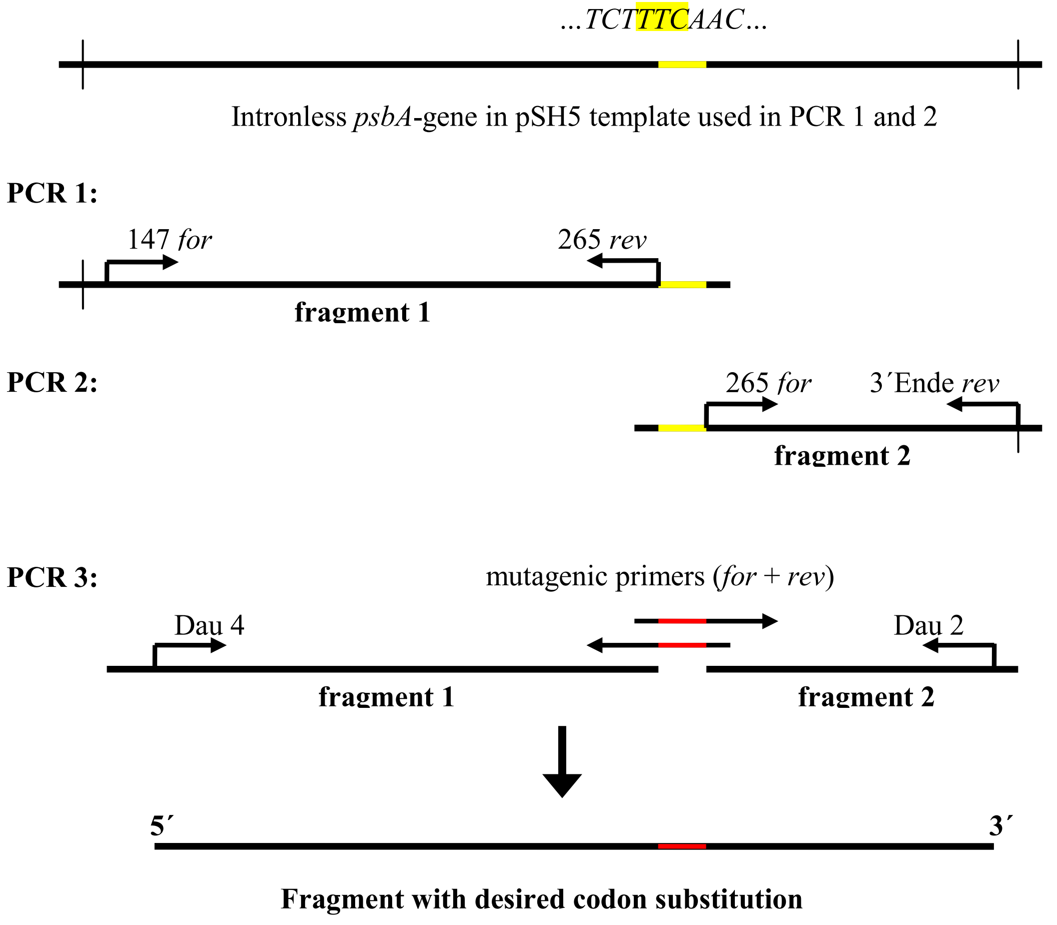

Table S1. Primers used for the site-directed mutagenesis.

A. Forward and reverse primers used in PCR1 and PCR2 for the generation of the DNA fragments 1 and 2, as indicated in the scheme of Figure S1.

Primer names	Nucleotide sequence in 5´→ 3´ direction
147 *for*	CATCGCTTTCATCGCTGCTCC
265 *for*	AACAACTCTCGTTCATTACACTTC
265 *rev*	AGAAGCGTATTGGAAGATTAGAC
3´Ende *rev*	GGGACGTCCTGCCAACTGCCTATGG
Dau 4	GGGTCGTGAGTGGGAATTATCTTTCCG
Dau 2	GCTAGAGTTAGTTGAAGCTAAGTCTAGAGGGA

B. Mutagenic primers used to introduce threonine (Thr) and serine (Ser) on the place of phenylalanine at position 265. The codons for Thr and Ser are highlighted in grey.

**Mutant**	**Primer names**	**Nucleotide sequence in 5′→ 3′ direction**
F265T	265 Thr *for*<br>265 Thr *rev*	CCAATACGCTTCTACAAACAACTCTCGTTCATTACACTTC<br>GAACGAGAGTTGTTTGTAGAAGCGTATTGGAAGATTAGAC
F265S	265 Ser *for*<br>265 Ser *rev*	CCAATACGCTTCTTCAAACAACTCTCGTTCATTACACTTC<br>GAACGAGAGTTGTTTGAAGAAGCGTATTGGAAGATTAGAC

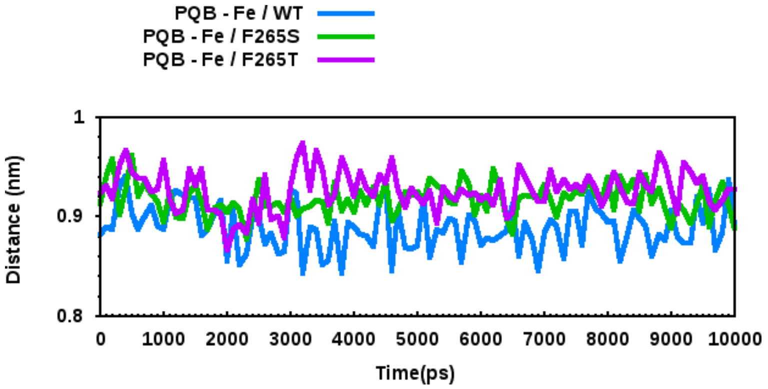

Fig. S3. Distance between the non-heme iron ion and the centre of mass of the ring moiety of Q_B as a function of time. Fluctuations from 8.6 to 9.2 Å with an average distance of about 9.0 Å were observed during the simulation of the WT complex. The distance between the centre of mass of the quinone ring and the non-heme iron atom during the simulation of the F265T mutant varied from about 8.6 to 9.8 Å, while in the case of the mutant F265S the same distance varied from about 8.8 to 9.6 Å, with an average value of about 9.2 Å. It is noteworthy that after about 5 ns of the simulations of the mutated systems the position of the ligand with respect to the non-heme iron atom remained stable.

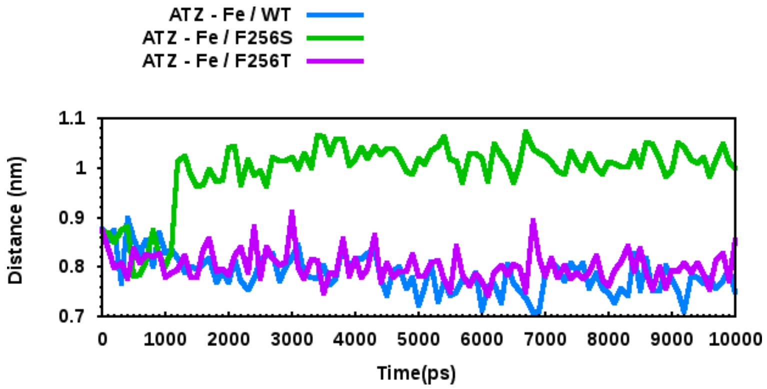

Fig. S4. Distance between the non-heme iron ion and the centre of mass of the triazine ring as a function of time. At the beginning of the simulations the distance between the centre of mass of the ring moiety of ATZ and the iron atom was about 8.8 Å for the WT complex and about 8.7 Å for the F265T and the F265S mutant complexes. During the first nanosecond of the three simulations ATZ changed its position in a similar way; only a small decrease in the distance between the centre of mass of the triazine ring and the non-heme iron atom was observed. During the following 9 ns of the simulations the position of the ATZ molecule within the WT complex and the complex with the F265T mutation was stable. The position of the ATZ molecule within the WT complex remained within the 7.0-8.5 Å range distance, while within the complex with the F265T mutation varied between 7.5 and 9.0 Å. During the simulation of the F265S mutant complex ATZ significantly changed its position at the beginning of the second nanosecond (at about 1.2 ns), so that the distance between the non-heme iron and the centre of mass of the triazine ring reached 10.1 Å. Over the following 8 ns of the simulation ATZ remained within a distance of about 9.7- 10.7 Å to the iron atom.

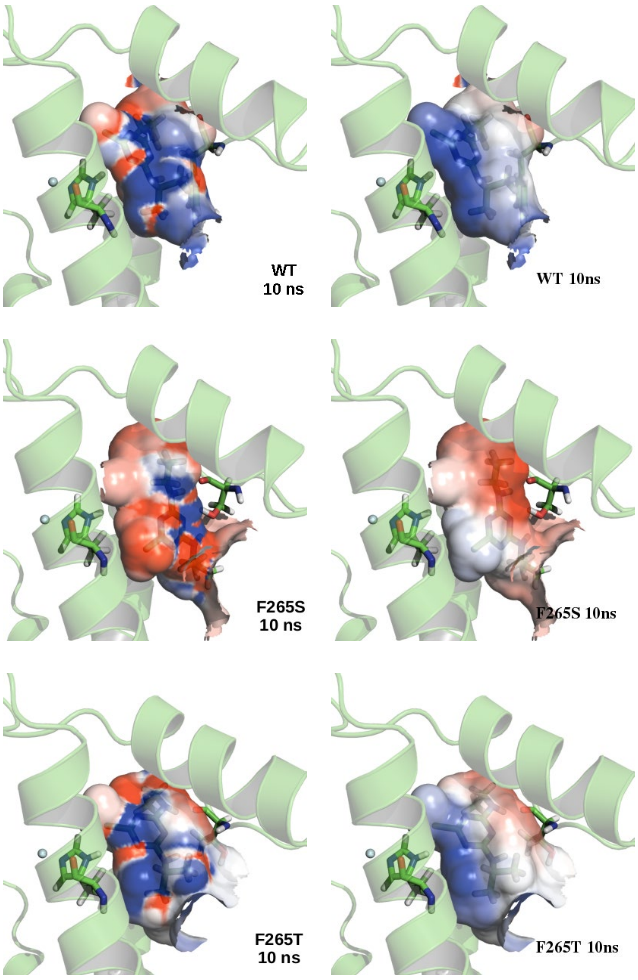

Fig. S5. Snapshots representing the surface of the protein D1 around the ATZ molecule coloured by electrostatic potential values. The colour scale is from -10 kT/e (red) to +10 kT/e (blue). Left panels– all atomic charges are considered during the calculations; right panels – atomic charges of ATZ set to zero.

A **B** **C**

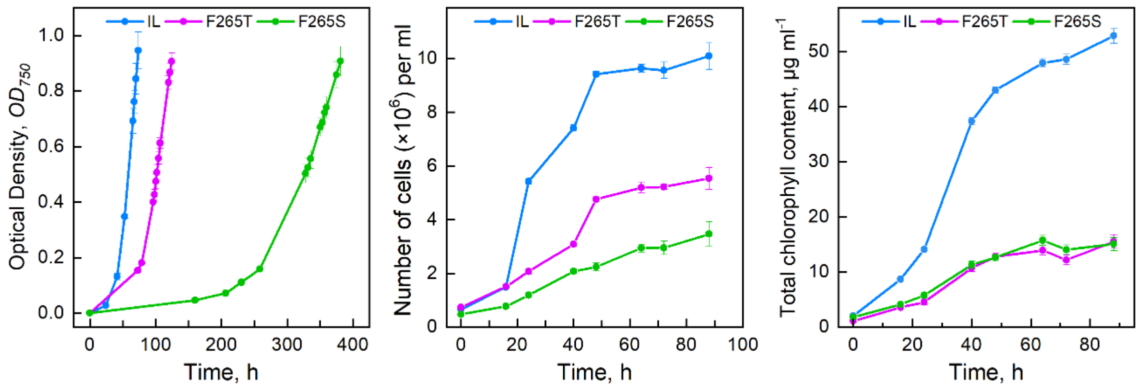

Fig. S6. Growth curves of the reference strain IL and the two D1 mutants. (**A**) Curves of photoautotrophic growth of the *Chlamydomonas* strains grown in high salt medium bubbled with 2.5% CO_2 at 23 °C and illumination of 80 µmol m^{-2} s^{-1} measured as optical density (*OD_{750}*). Curves of mixotrophic growth of the *Chlamydomonas* strains grown in TAP medium, at 24 °C, illumination of 50 µmol m^{-2} s^{-1} and 150 rpm agitation measured as cell number per ml (**B**) and total chlorophyll content (**C**). Each point represents an average of 2-3 independent experiments, ±SE (n=2-3).

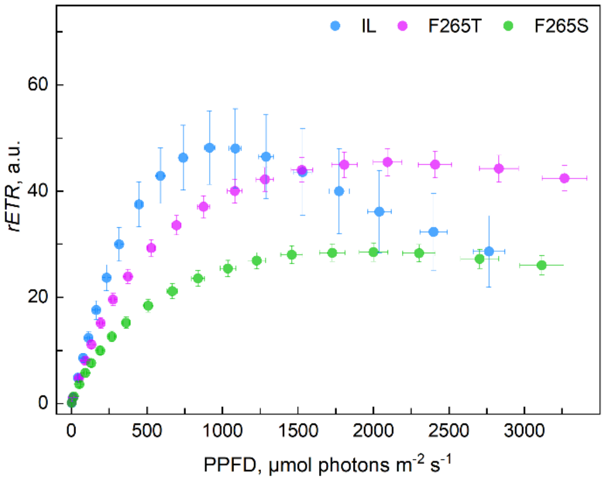

Fig. S7. Rapid light curves of relative electron transfer rate (*rETR*) of the three *Chlamydomonas* strains. The measurements were performed after 30 s exposure of the samples at the indicated light intensities. Each point represents an average of at least four independent experiments, ±SE (n=4-7).

Table S2. Parameters calculated from the rapid light curves of the *rETR* of PSII of the three *Chlamydomonas* strains. $rETR_{max}$ is the maximum value for *rETR*; the photosynthetic quantum efficiency (α, μmol electrons/photons) is calculated from the initial slop of the RLC; the minimum saturating irradiance is calculated as $E_k=ETR_{max}/\alpha$; E_{max} is the irradiance at which the ETR_{max} was reached.Average values from at least four independent experiments, ±SE (n=4-7).

Strains	$rETR_{max}$	Photosynthetic quantum efficiency	E_k	E_{max}
IL	56±7	0.11±0.009	515±30	1107±100
F265T	46±3	0.09±0.006	498±5	2015±71
F265S	29±2	0.06±0.004	469±7	1961±79

The $rETR$ was calculated as:

$$rETR = Y(II) \times PPFD \times ETR_{Factor} \times 0.5, \quad \text{(Eq. 1)}$$

where *PPFD* is that of the actinic light; ETR_{Factor} is the estimated fraction of incident photons absorbed by PSII, measured by an integrating sphere, and multiplied by 0.5, assuming equal distribution of the absorbed energy between PSII and PSI, and *Y(II)* represents the estimated effective quantum yield of PSII photochemistry under illumination, calculated as:

$$Y(II) = (F_m' - F_s')/F_m', \quad \text{(Eq. 2)}$$

where F_m' is the maximum fluorescence measured under illumination and F_s is the steady-state fluorescence. The average calculated ETR_{Factor} of IL was 0.36±0.02 (n=14), ETR_{Factor} of F265T was 0.42±0.03 (n=8) and ETR_{Factor} of F265S was 0.49±0.02 (n=8). The RLC parameters were calculated after the application of ETR_{Factor} to each single experimental curve as previously described (Serôdio *et al.*, 2006): ETR_{max} – is the maximum value for *rETR*; the photosynthetic quantum efficiency (α) is estimated from the initial slope of the RLC (first 5 points, average max PPFD ≤ 130 μmol photons m^{-2} s^{-1}); the minimum saturating irradiance is calculated as ETR_{max}/α.

Table S3. Characteristics of the temperature dependency of fluorescence relaxation in *Chlamydomonas* strains measured in presence of 20 μM DCMU. The multicomponent deconvolution of the curves of the fluorescence decay was performed according to the equation $F_{(t)}=ae^{-bt}+ce^{-dt}$, where F is the normalized fluorescence intensity, t is time and a, b, c, and d are constants, using standard fitting procedures of Sigma Plot (SYSTAT Software).

Strain	T, °C	A_{fast}	τ_{fast} (ms)	A_{slow}	τ_{slow} (s)
IL	15	0.31	229	0.69	2.33
	20	0.38	186	0.62	1.69
	**25**	**0.43**	**115**	**0.57**	**1.00**
	30	0.47	79	0.53	0.65
F265T	15	0.25	156	0.75	2.17
	20	0.27	125	0.73	1.25
	**25**	**0.40**	**100**	**0.60**	**1.01**
	30	0.37	74	0.63	0.61
F265S	15	0.26	191	0.74	2.43
	20	0.35	185	0.65	1.69
	**25**	**0.36**	**108**	**0.64**	**0.96**
	30	0.36	75	0.64	0.59

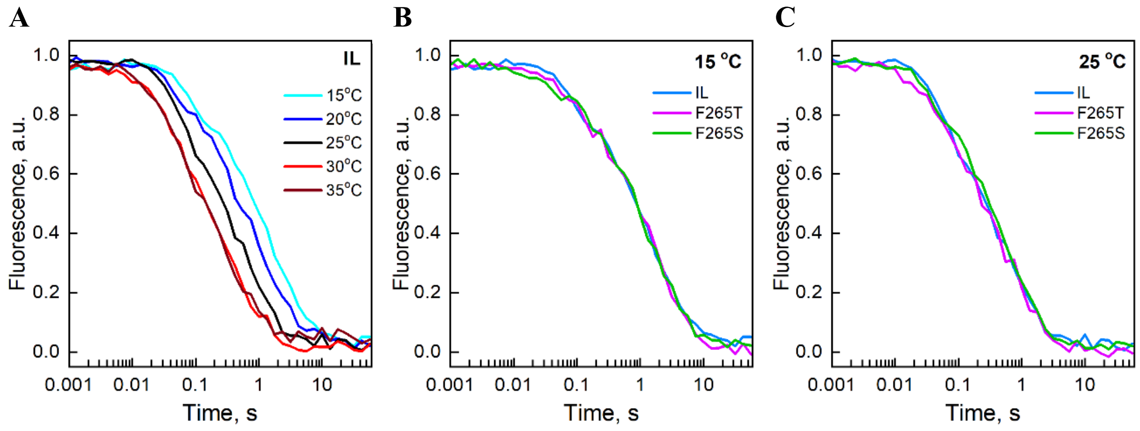

Fig. S8. Fluorescence relaxation kinetics in *Chlamydomonas* strains measured in the presence of 2×10^{-5} M DCMU. (**A**) Temperature dependency of fluorescence relaxation in IL strain; (**B**) and (**C**) fluorescence relaxation kinetics in IL, F265T and F265S strains measured at 15 °C and 25 °C, respectively.

Table S4. Activation energies and pre-exponential factors of Q_A^- reoxidation in the IL strain and the F265T and F265S mutants of *C. reinhardtii*. Data from Fig. S8 were fitted with a two-exponential model (Table S3) and the resulting rate constant values (k) were fitted with the Arrhenius equation ($k=s_0\exp(-Ea/k_bT)$, where k_b is Boltzmann's constant and T is temperature) to obtain values of the activation energy (Ea) and preexponential factor (s_0).

**Strain**	**PSII centres with fast reoxidation of Q_A^- comprising 25-43 % of PSII**		**PSII centres with slow reoxidation of Q_A^- comprising 57-75 % of PSII**	
	Ea (meV)	s_0 (s^{-1})	Ea (meV)	s_0 (s^{-1})
**IL**	552	1.85×10^{7}	655	1.16×10^{11}
**F265T**	370	1.86×10^{4}	605	1.85×10^{10}
**F265S**	501	2.73×10^{6}	724	1.78×10^{12}

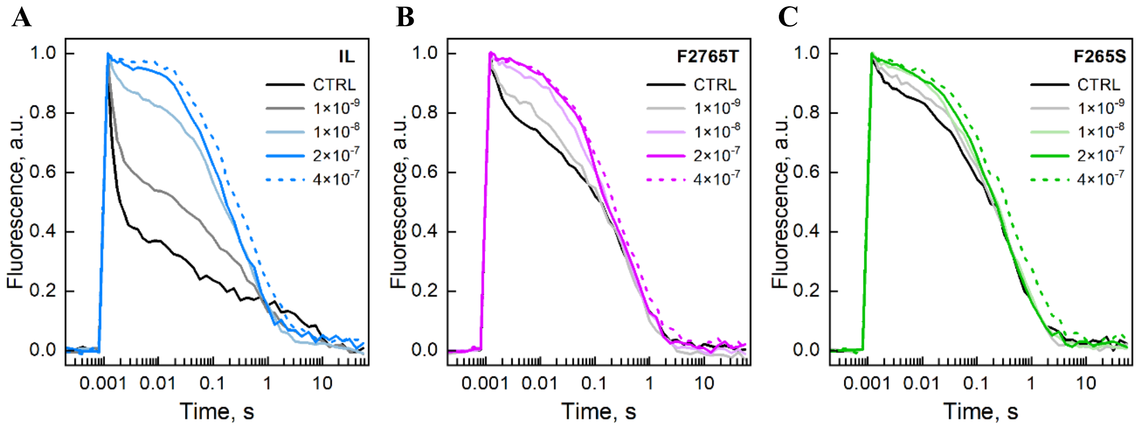

Fig. S9. Effect of ATZ on Q_A^- reoxidation kinetics of *Chlamydomonas* strains. Chlorophyll *a* fluorescence decay after a single turnover flash in IL (**A**), F265T (**B**) and F265S (**C**) was recorded at room temperature and increasing herbicide concentration in the range from 1×10^{-9} M to 4×10^{-7} M ATZ.

References

Dauvillee D, Hilbig L, Preiss S, Johanningmeier U. (2004) Minimal extent of sequence homology required for homologous recombination at the *psb*A locus in *Chlamydomonas reinhardtii* chloroplasts using PCR-generated DNA fragments. *Photosynthesis research* 79: 219-224.

Rea G, Lambreva M, Polticelli F, Bertalan I, Antonacci A, Pastorelli S, Damasso M, Johanningmeier U, Giardi MT, Herrera-Estrella A. (2011) Directed evolution and in silico analysis of reaction centre proteins reveal molecular signatures of photosynthesis adaptation to radiation pressure. *PLoS ONE* 6: e16216.

Serôdio J, Vieira S, Cruz S, Coelho H. (2006) Rapid light-response curves of chlorophyll fluorescence in microalgae: Relationship to steady-state light curves and non-photochemical quenching in benthic diatom-dominated assemblages. *Photosynthesis Research* 90: 29-43.
